# Modulation of Dengue/Zika Virus Pathogenicity by Antibody-Dependent Enhancement and Strategies to Protect Against Enhancement in Zika Virus Infection

**DOI:** 10.3389/fimmu.2018.00597

**Published:** 2018-04-23

**Authors:** Rekha Khandia, Ashok Munjal, Kuldeep Dhama, Kumaragurubaran Karthik, Ruchi Tiwari, Yashpal Singh Malik, Raj Kumar Singh, Wanpen Chaicumpa

**Affiliations:** ^1^Department of Biochemistry and Genetics, Barkatullah University, Bhopal, India; ^2^Division of Pathology, ICAR-Indian Veterinary Research Institute, Bareilly, India; ^3^Central University Laboratory, Tamil Nadu Veterinary and Animal Sciences University, Chennai, India; ^4^Department of Veterinary Microbiology and Immunology, College of Veterinary Sciences, Uttar Pradesh Pandit Deen Dayal Upadhyaya Pashu Chikitsa Vigyan Vishwavidyalaya Evam Go-Anusandhan Sansthan (DUVASU), Mathura, India; ^5^Division of Biological Standardization, ICAR-Indian Veterinary Research Institute, Bareilly, India; ^6^ICAR-Indian Veterinary Research Institute, Bareilly, India; ^7^Center of Research Excellence on Therapeutic Proteins and Antibody Engineering, Department of Parasitology, Faculty of Medicine SIriraj Hospital, Mahidol University, Bangkok, Thailand

**Keywords:** antibody-dependent enhancement, Dengue virus, Zika virus, pathogenesis, counteracting strategies, engineered antibodies

## Abstract

Antibody-dependent enhancement (ADE) is a phenomenon in which preexisting poorly neutralizing antibodies leads to enhanced infection. It is a serious concern with mosquito-borne flaviviruses such as Dengue virus (DENV) and Zika virus (ZIKV). *In vitro* experimental evidences have indicated the preventive, as well as a pathogenicity-enhancing role, of preexisting DENV antibodies in ZIKV infections. ADE has been confirmed in DENV but not ZIKV infections. Principally, the Fc region of the anti-DENV antibody binds with the fragment crystallizable gamma receptor (FcγR), and subsequent C1q interactions and immune effector functions are responsible for the ADE. In contrast to normal DENV infections, with ADE in DENV infections, inhibition of STAT1 phosphorylation and a reduction in IRF-1 gene expression, NOS2 levels, and RIG-1 and MDA-5 expression levels occurs. FcγRIIA is the most permissive FcγR for DENV-ADE, and under hypoxic conditions, hypoxia-inducible factor-1 alpha transcriptionally enhances expression levels of FcγRIIA, which further enhances ADE. To produce therapeutic antibodies with broad reactivity to different DENV serotypes, as well as to ZIKV, bispecific antibodies, Fc region mutants, modified Fc regions, and anti-idiotypic antibodies may be engineered. An in-depth understanding of the immunological and molecular mechanisms of DENV-ADE of ZIKV pathogenicity will be useful for the design of common and safe therapeutics and prophylactics against both viral pathogens. The present review discusses the role of DENV antibodies in modulating DENV/ZIKV pathogenicity/infection and strategies to counter ADE to protect against Zika infection.

## Introduction

Antibody-dependent enhancement (ADE) is a phenomenon in which the intensity of infection increases in the presence of preexisting poorly neutralizing antibodies. Dengue virus (DENV) and Zika virus (ZIKV) are positive-stranded RNA viruses in the family *Flaviviridae*. Both viruses infect humans, result in similar clinical symptoms, and are transmitted mainly by mosquitoes (1, 2). The high public health concerns of ZIKV have attracted attention of worldwide researchers for designing and developing effective vaccines, drugs, therapeutics along with formulating appropriate prevention, and control strategies to counter this emerging pathogen (3, 4). ZIKV has only one serotype (5), whereas DENV comprises four recognized serotypes (DENV1–DENV4). The envelope (E) and non-structural 1 (NS1) proteins of ZIKV and DENV share sequence similarity in the range of 54.0–57.8% (6, 7). The similarity in E proteins of the four different DENV serotypes is 63%, which is slightly higher than the similarity between ZIKV and DENV (8). This sequence similarity results in a cross-reactive antibody response between ZIKV and DENV, which has been recently described (9, 10). Extensive cross-reactivity between ZIKV and DENV virus antibodies may not only create problems for antibody-based diagnosis (11) but also pose challenges because of the possibility of ADE of ZIKV infection resulting from DENV non-neutralizing antibodies as demonstrated in many *in vitro* experiments. Antibodies to DENV have been reported to interact with ZIKV, producing both protective effects (owing to cross-neutralization) (12, 13) and ADE (owing to poorly neutralizing antibodies) (14, 15). ADE of DENV infections has been observed in young children born to mothers’ who are immune to DENV. Catabolism of maternal antibodies in the child results in sub-neutralizing concentrations of antibody, which can lead to ADE (16). DENV-ADE of ZIKV infection has been well established in *in vitro* and in laboratory animal models; however, this is not the case in experiments with monkeys, in which preexisting DENV antibodies did not worsen ZIKV infection. In fact, in comparison with non-exposure, pre-exposure to DENV helped in clearing ZIKV infection more quickly in monkeys (13). Investigation of a cohort of patients, who were previously infected with DENV showed neither ADE of ZIKV infection nor significant differences in ZIKV loads (17). Reciprocally, antibodies to ZIKV have been shown to enhance DENV2 viremias in a rhesus macaque model that exhibits neutropenia, lymphocytosis, hyperglycemia, and higher reticulocyte counts, with the involvement of inflammatory mediators (18).

The phenomenon of ADE has been well documented and reproduced using both monoclonal antibodies and polyclonal sera in *in vitro* models using Fcγ receptor-expressing cells, including the cell lines K562 and U937, as well as primary human monocytes, macrophages, and dendritic cells. During ADE in THP-1 cells, type-I interferon (IFN) is suppressed, and IL-6 and IL-10 levels are increased (19, 20). ADE has been difficult to reproduce in animal models, as DENV replicates poorly in animals other than humans (approximately 1,000 times less) and severe disease outcomes such as dengue hemorrhagic fever (DHF) are not observed. IFN receptor-deficient AG129 mice, commonly used in ADE studies, display neurological symptoms and splenomegaly (21), and develop lethal vascular leakage and other symptoms similar to DHF. In non-human primates, ADE is difficult to reproduce as DHF and dengue shock syndrome (DSS) occur infrequently. However, there is little evidence for the enhancement of viremia in animals treated with below neutralizing levels of DENV antibody relative to that in controls (22). In humans also, not all studies support the existence of ADE. In a study by Libraty et al. (23)., infants born to DENV3-immune mothers showed ADE in subsequent DENV3 infections, but a higher level of DENV3 ADE activity was not associated with DHF. In contrast, in another study, infants born to DENV2-immune mothers exhibited enhanced infections and DHF/DSS of less than 12 months of age (24). The present review focuses mainly on DENV-ADE and the role of DENV antibodies in possible ZIKV ADE. In addition, the role of FcγRs and different molecular events in ADE like inhibition of STAT1, IFN signaling, nitric oxide (NO) production, retinoic acid-inducible gene (RIG)-1, and melanoma differentiation-associated gene (MDA)-5 gene expression, as well as the roles of IL-10 upregulation, autophagy, and hypoxia in ADE are discussed. This review describes *in vitro* and *in vivo* experiments on ADE in various models, including mice, macaque, and humans, and the use of mAbs, polyclonal sera, or human cord blood sera in various experiments. DENV antibodies play a role in modulating immune responses, i.e., cross-protecting or causing ADE in DENV or ZIKV infection. We discuss antibody engineering, including the introduction of mutations like LALA in the Fc region, and the use of bispecific antibodies, swapping of Fc regions, and production of anti-idiotypic antibodies as some strategies that can be employed to reduce/prevent ADE by eliminating strong interactions between the Fc region of antibodies and FcγR and inhibiting antibody effector functions. These strategies may be helpful in the development of prophylactics.

## The Antibody-Dependent Enhancement (ADE)

The phenomenon of ADE in arbovirus infections was first described in 1964 (25). Prior to this, there were reports that preexisting non-neutralizing antibodies were responsible for enhanced infection with several human and animal viruses, including DENV (26), ZIKV (27), Ebola virus (28), HIV (29), Aleutian mink disease parvovirus (30), coxsackievirus B virus (31), equine infectious anemia virus (32), feline infectious peritonitis virus (33), simian hemorrhagic fever virus (34), caprine arthritis virus (35), porcine reproductive and respiratory syndrome virus (36), and African swine fever virus (37). Similarly, in the case of Murray Valley encephalitis (MVE) virus, higher antibody concentrations resulted in virus neutralization and reduced plaque numbers in chicken embryo (CE) cell monolayers, but with lower concentrations of antibodies, the number of plaques increased (38). Halstead et al. (39) reported that previous DENV infection predisposed humans to more severe DENV disease, and the role of ADE was hypothesized in causing severe dengue in children with secondary DENV infections. ADE was observed *in vitro*, with peripheral blood leukocytes (PBLs) from DENV-immune rhesus macaques enhancing the growth of virus. In PBLs from non-immunized animals, DENV decay was similar to decay in a cell-free system, whereas the growth curves of DENV with PBLs from immunized animals were similar to the growth curve of other susceptible cells. This indicated the enhancement of DENV infection in PBLs in the presence of preexisting antibodies. The indirect evidence for preexisting antibodies playing a role in ADE was that PBL permissiveness to DENV was associated with the development of a humoral immune response and could be reproducibly demonstrated only in PBLs from immune donors (40). ADE was also demonstrated in rhesus monkeys, a natural DENV host, which received intravenous injections of human cord blood sera from normal or DENV-immune mothers, and higher and longer viremias were observed in animals receiving DENV-immune cord blood sera in comparison with their paired controls who received cord blood serum from non-immune donors (41).

In DENV-ADE, non-neutralizing antibodies form an immunocomplex with DENV, and this complex is internalized by Fc-receptor-bearing cells, including monocytes/macrophages and dendritic cells, culminating in an increased virus load and disease severity. A few studies have suggested that virus immune-complex-mediated suppression of intracellular antiviral responses is responsible for enhanced virus replication (19, 42), with some suggesting that increased entry of DENV through FcγR (43) or increased fusion potential of the virus (44) is behind the phenomenon of ADE.

## Molecular Mechanisms Underlying DENV-ADE

During ADE, an equal number of intracellular viral RNA copies were found in DENV and DENV-ADE-infected K562 cells (45). K562 cell is a monocytic cell line that lack type-I IFN genes; therefore, ADE in this model is independent of IFN suppression. During DENV-ADE, early suppression of NOS2 synthesis helps the virus to evade early innate immune detection. RIG-1 and MDA-5 protein expression is downregulated, whereas that of IL-10 and autophagy-related proteins such as DAK and Atg5–Atg12 is upregulated. Molecular mechanisms underlying the phenomenon of ADE have mostly been explored in *in vitro* studies on DENV. In one *in vivo* experiment, Ng et al. (46) showed increased severity of dengue disease in AG129 mice (IFN-α/β/γ receptor deficient), with DENV2-infected mice born to DENV1-immune mothers dying and displaying higher levels of IL-6 and TNF-α than mice born to naive mothers. The possible mechanisms involved in ADE of DENV infections are outlined below.

(a) *Inhibition of STAT1 and interferon signaling*: an antiviral response is induced upon infection with viruses. Type-I IFNs stimulate IFN receptor subunits IFNAR1 and IFNAR2 to activate Janus kinases Jak1 and Tyk2 and signal transducers of transcription STAT1 and STAT2, which upregulate many IFN-stimulated genes (47). IFN antagonism is an essential requirement for mosquito-borne viruses to maintain high viral loads in the blood and to continue the vector–host cycle. Inhibition of IFN signaling is mediated mainly by the NS5 protein. NS5 proteins in flaviviruses exhibit functional similarities and inhibit IFN signaling through various mechanisms. In WNV infections, NS5 targets the host protein prolidase to inhibit the expression of IFNAR1 (48); whereas in DENV infections, NS5 recruits the host ubiquitin protein ligase E3 component N-recognin (UBR4) to degrade STAT2 (49). In contrast, ZIKV has been shown to bind to and reduce STAT2 levels by dose-dependent proteosomal degradation (50).
  In THP-1 cells, STAT1 phosphorylation increased during DENV infection, whereas, in the case of DENV-ADE, STAT1 phosphorylation was inhibited. Similarly, IRF-1 gene expression is increased in DENV infections but reduced with DENV-ADE (51). ADE also enhances the number of fusion-positive cells and number of fusions per cell, resulting in increased internalization of virus. However, contrary to the results of other experiments, DENV2-ADE was not shown to suppress IFNs; rather, it prevented induction (44). Therefore, any increase in the amount of virus remains unnoticed initially, eventually allowing viral replication to higher titers.
(b) *Inhibition of NO expression*: NO synthesized by nitric oxide synthase 2 (NOS2), part of an innate immune response, inhibits DENV viral RNA synthesis through attenuating the RNA-dependent RNA polymerase (52). When NO levels were evaluated at different time intervals in DENV and DENV-ADE-infected cells, direct DENV infection was found to increase NOS2 levels, whereas DENV-ADE-infected cells showed decreased levels of NOS2. This result suggested initial evasion of innate immunity. In a study of 60 patients with primary DENV fever, primary DENV hemorrhagic fever, secondary DENV fever, and secondary DENV hemorrhagic fever, mean NO levels in plasma were determined. The highest viremia and lowest NO plasma levels were observed in the secondary DENV hemorrhagic fever group. NO production was not suppressed in patients with primary DENV hemorrhagic fever, suggesting a role of ADE in NO suppression (51).
(c) *Inhibition of RIG-1 and MDA-5 genes*: RIG-1 and MDA-5 genes participate in recognition of viral RNA and induce type-I IFN signaling through mitochondria antiviral protein (MAVS) (53). RIG-1 recognizes small RNAs with 5′-triphosphate caps, whereas MDA-5 recognizes long genomic RNA and replication intermediates (54). The levels of RIG-1 and MDA-5 increased in DENV-infected THP-1 cells relative to those that were uninfected; in contrast, they decreased in DENV-ADE infections. In DENV infections, expression of signaling molecules downstream of RIG-1/MDA-5 results in type-I IFN production. DENV-ADE complexes did not stimulate the expression of RIG-1/MDA-5 signaling molecules, and type-I IFN production was attenuated (19). In peripheral blood mononuclear cells (PBMCs) from patients with secondary DENV hemorrhagic fever, RIG-1, MDA-5, and IFN-β promoter stimulator (IPS)-1 were significantly suppressed in comparison with that in PBMCs of patients with DENV fever only. NF-κB is a heteromdimer consisting of p65 and p50 subunits, associated with inhibitor protein Iκ-B. Lower RIG-1 and MDA-5 decrease the NF-κB levels, which manifest into increase in the expression of subunit p65 of NF-κB and decrease in Iκ-B. NF-κB is a transcription factor, which regulates the expression of pro-inflammatory cytokines including TNF-α. Iκ-B degradation leads to the translocation of NF-κB to the nucleus and it drives expression of pro-inflammatory cytokines (55).
  Interferon-β levels were reduced in PBMCs from patients with secondary DENV hemorrhagic fever relative to those in PBMCs from patients with DENV fever. IRF-1 transcription was also reduced in DENV-ADE-infected K562 cells (45). In THP-1 cells, IL-6 is upregulated, whereas IL-12 and IFN-γ are downregulated, which indicates that there is a bias for Th2-type response. During natural DHF/DSS in infants, significantly higher levels of TNF-α have been observed in comparison to healthy controls and blocking of TNF-α has shown to reduce the mortality (56). In U937-derived macrophages, a higher level of TNF-α has been observed and the increased concentrations of pro-inflammatory cytokines (TNF-α, IL-6, IL-1β, IL-8) are linked with the fatal plasma leakage associated with the severe form of DENV infection. During ADE of infection, both the IL-10 as well as IL-6 levels are increased (51).
(d) *Upregulation of IL-10*: in DENV infection *via* the Fc receptor pathway, ADE modifies not only the entry of opsonized virus into cells but also innate and adaptive intracellular immune response. This facilitates the production of anti-inflammatory cytokine IL-10, which decrease the expression of IL-12 and IFN-γ (51). IL-10 also mediates SOCS-3 activation; PBMCs from patients with secondary DENV infection shown to express higher levels of SOCS-3 than those from patients with primary DENV infections (19). IL-10 production rapidly increased during DENV-ADE infections, and cell-type specificity and genetic polymorphisms of hosts affect IL-10 production (57). In a cohort of 45 Cuban patients, sera were found to have higher IL-10 levels during secondary DENV infections (58). Dendritic cells, B cells, mast cells, monocytes, and NK cells have shown ADE-dependent production of IL-10. Patients with secondary DENV2 infections exhibited significantly higher IL-10 levels in a study on 182 patients hospitalized with dengue in Taiwan (59). In contrast, in China, of 353 patients hospitalized with dengue (212 patients with primary and 141 with secondary DENV1 infections), no statistically significant difference was observed in IL-10 levels in patients with primary and secondary infections (60). During early infections [2 hours post infection (hpi) and 24 hpi], upregulation of IFN-β, TNF-α, IL1β, and IL-6 cytokines, with little to no IL-10, was observed in DENV-infected primary human macrophages, despite ADE. Of note, IL-10 plays an important role in intrinsic ADE in monocytes (51), whereas, in macrophages, IL-10 is poorly induced after ADE (20).
(e) *Upregulation of autophagy*: autophagy contributes to cell survival during nutrient starvation, infection, and environmental and cellular stress conditions. During autophagy, cytoplasmic or cellular organelles are enclosed in double membranous vesicles called autophagosomes. Later, these autophagosomes fuse with lysosomes to form autophagolysosomes, in which cellular components are digested. Autophagy exerts both pro-viral and anti-viral effects (61). DENV infection may promote autophagy and the formation of the autophagosomes, amplifying DENV titers in an Atg5-dependent manner. DENV replication was suppressed when autophagy was blocked with authophagy inhibitor 3-methyladenine (3-MA) (62). When infected with ZIKV, autophagy is increased in human cytotrophoblast cells (JEG-3 cell line), as evidenced by the accumulation of LC3-II in ZIKV-infected cells. The use of 3-MA, chloroquine (CQ), and Baf A1, all of which inhibit autophagy at various points, decreased ZIKV titers, whereas administration of autophagy inducers like rapamycin and Torin 1 resulted in increased ZIKV titers in trophoblasts of pregnant mice. ATG16L1 plays an essential role in autophagosome maturation by directing LC3 to autophagosomes to facilitate fusion with lysosomes. The placenta of mice hypomorphic for gene Atg16L1 (Atg16l1HM) showed an approximately 10-fold decrease in ZIKV titers in comparison with placentas from wild-type mice (63). In suckling mice infected with DENV2, the use of autophagy inducer rapamycin increased titers, whereas autophagy 3-MA decreased titers, in the brains of infected mice (64). The autophagic machinery also blocks IFN-β production (65), and a DENV non-structural protein, NS4A, uniquely upregulates autophagy. In the case of DENV-ADE, autophagy is upregulated in a PI3K-dependent manner (66).
  In DENV-ADE infection, autophagy was significantly increased in pre-basophil-like KU812 cells and immature mast cell-like HMC-1 cells. NS4A and NS4B of ZIKV suppress host Akt-mTOR signaling, leading to increased autophagy for increased viral replication and impaired neurogenesis of human fetal neural stem cells (fNSCs) (67), whereas DENV NS4A alone can upregulate autophagy in epithelial cells (66).
  Dengue virus-ADE infection also showed an increase in the number of autophagosome vesicles, LC3 punctuation, LC3-II accumulation, and p62 degradation (68). Ubol and Halstead (69) showed that, in the presence of DENV-ADE in humans, negative regulators of RIG-1 and MDA-5 (DAK and Atg5–Atg12) were activated, disrupting RIG-1/MDA-5 signaling and limiting the type-I IFN-mediated antiviral response.
  The impact of autophagy on flavivirus infections is possibly species specific. In addition, it is dependent partly on factors such as the cell line, virus strain, and autophagy inducer/inhibitors used in the study.
(f) *Role of FcγRs in ADE*: FcγR generally participates in phagocytosis. Antibody-opsonized DENV enters a cell through a different pathway than non-opsonized DENV. At dilutions higher than neutralizing end points, immune complexes are formed between DENV and the antibody, which binds to the cell receptor through the Fc region to be internalized. This facilitates viral infection and a subsequent increase in virus production (70). Entry of both DENV and antibody-opsonized DENV in P388D1 cells is dynamin, actin, pH, and Rab7 dependent. DENV entry is independent of FcγR, PI3K, and Rab5; on the other hand, infection with antibody-opsonized DENV requires all these (71). FcγR are strongly expressed by monocytes, macrophages, and dendritic cells, when these cells are infected with opsonized viruses. Through FcγRs, IgG antibodies are capable of mediating ADE infection. FcμR is rare in the phagocytes of mice and humans (72); therefore, the involvement of IgM in ADE may be negligible. However, when FcγRs are engaged in binding to immunocomplexes, instead of activating an immune response, innate immunity is suppressed by an increase in IL-10 production and Th2 bias, which eventually leads to an increase in virus output from the infected cells (73).
  Secondary DENV infections result in ADE, which leads to vascular leakage and endothelial permeability (74). Pathological amounts of NS1 protein from DENV1, DENV2, DENV3, and DENV4 result in vascular leakage and endothelial dysfunction, along with the production of key inflammatory cytokines. Immunization of mice with NS1 of DENV2 protects mice from lethal DENV2 challenge or ADE. With other serotypes (DENV1, DENV3, DENV4), also NS1 immunization offered substantial protection from lethal DENV-induced vascular leakage syndrome in mice (75). Fcγ receptor-dependent (and also independent) mechanisms are involved in NS1 mAb-mediated protection, as evidenced by an experiment on congenic Fcγ receptor I- and III-deficient mice (76).
  Because of its higher expression, FcγRIIA appears to be the more permissive than FcγRI for DENV-ADE. Internalization by FcγRI or FcγRIIA ligands affects the permissiveness of the complex (42). FcγRI-mediated phagocytosis has been found to be negatively regulated by FcγRIIB (77). IgG antibodies may cross the placenta and be transported through the placental blood to fetuses. In a fetus, infection is mediated by the neonatal Fc receptor (FcRn) (78). A close association between infectivity and phagocytic activity has been reported (79). FcγRIIA-mediated phagocytosis of the DENV-immune complex is initiated by lipid raft-induced receptor accumulation, which leads to the initiation of signaling cascades by the cytoplasmic domain of Ig gene family tyrosine activation motif (ITAM) molecules. ITAM molecules are phosphorylated at tyrosine residues by Src family kinases. These phosphorylated ITAM molecules recruit Syk/ZAP-70 kinases and other effectors to initiate phagocytosis (80). In WNV, a close relative of DENV, the quantity of antibodies determines this initiation process. With fewer antibodies, a single opsonized particle enters through clathrin-coated pit; a specialized membrane structure, constituting polyhedral lattice of clathrin protein to facilitate the receptor mediated endocytosis. When the antibody concentration is higher, antibody-mediated aggregates of multiple viral particles are phagocytosed (81). Similar to DENV, in visceral leishmaniasis, a protozoan disease, ADE has been demonstrated (73). When the surface of amastigotes forms an immune complex with IgG, the immune complex is ligated to FcγR, which facilitates the production of higher levels of IL-10. *Leishmania* disease severity is directly proportional to the levels of IL-10 in the plasma of patients (82). In DENV also, higher viremias have been associated with higher levels of IL-10 and disease severity (83). In ADE, because antibodies are non-neutralizing and present at lower levels, DENV and ZIKV are likely to enter through coated pits.
(g) *Role of hypoxia in ADE*: the level of oxygen plays a role in monocyte trafficking to the lymph nodes and spleen and in viral pathogenesis. Hypoxia can inhibit some viruses, such as adenovirus, influenza virus, and simian virus 40, because these viruses target O_2_-rich tissues; in contrast, hepatitis C virus (HCV) and Sendai virus usually reside in tissues with lower O_2_ levels (84–86). DENV replicates mainly in endothelial cells, fibroblasts, myeloid-derived cells, and lymphocytes (87–89). DENV is transported from the site of infection to lymph nodes by dendritic cells (90). The oxygen content of the microenvironment in lymph nodes is lower than the atmospheric oxygen level (~0.5–4.5% O_2_ vs. ~20% O_2_) (91). It is already an established phenomenon that, at a lower O_2_ level, immunoregulatory and inflammatory response-associated genes are overexpressed (92). At a lower O_2_ level, hypoxia-inducible factor-1 alpha (HIF1α) may act as a transcriptional enhancer that induces the expression of FcγRIIA. As result of FcγRIIA expression, internalization of DENV-immune complexes increases (93). In hypoxic conditions, opsonization of DENV by antibodies results in the accumulation of DENV particles. Thus, *in vitro* assays to determine the protective, neutralizing, or enhancing potential of antibodies against DENV or ZIKV that are conducted at higher O_2_ concentrations might lead to an underestimation of ADE potential *in vivo* (93). Evidence of the role of hypoxia in *in vivo* experiments is indirect (94) and has shown the upregulation of genes (PRKCB1, ARF6, PRKCD, PXN, FYN, VAMP3, DGKB, DOCK1, PTEN) involved in FcγR-mediated phagocytic signaling in mice cerebellums under hypoxic conditions.
(h) *Relationship between lipid rafts and ADE*: cholesterol plays a role in replication of various flaviviruses, and cholesterol metabolism is required for replication of the DENV genome (95). The major target of DENV is cells of the monocyte lineage that express FcγRs (96). Binding of IgG with lipid raft-associated FcγRs is essential for Src family kinase-mediated signaling (97), which contributes to cellular activation, cytotoxicity, and internalization of IgG-opsonized virus particles (98). For some viruses, proteins in lipid rafts act as receptors for viral entry (99). To determine the role of lipid rafts in ADE of DENV, a study was conducted with the drugs filipin, nystatin, and MβCD, which inhibit the formation of lipid rafts in U937 cells. FcγR associates with lipid rafts upon binding with IgG. These rafts were found to be an essential requirement for DENV-ADE, since inhibition of lipid raft formation reduces ADE (100). The association between the immune complexes and FcγRII formed during ADE is not stable in the absence of lipid rafts; thus, dissociation of these complexes occurs (101). Distortion of lipid rafts also prevents signaling essential for the internalization of immune complexes. Thus, drugs that inhibit lipid raft formation, such as nystatin and filipin (102), may lead to the inhibition of ADE caused by DENV (101).

**Figure 1:**
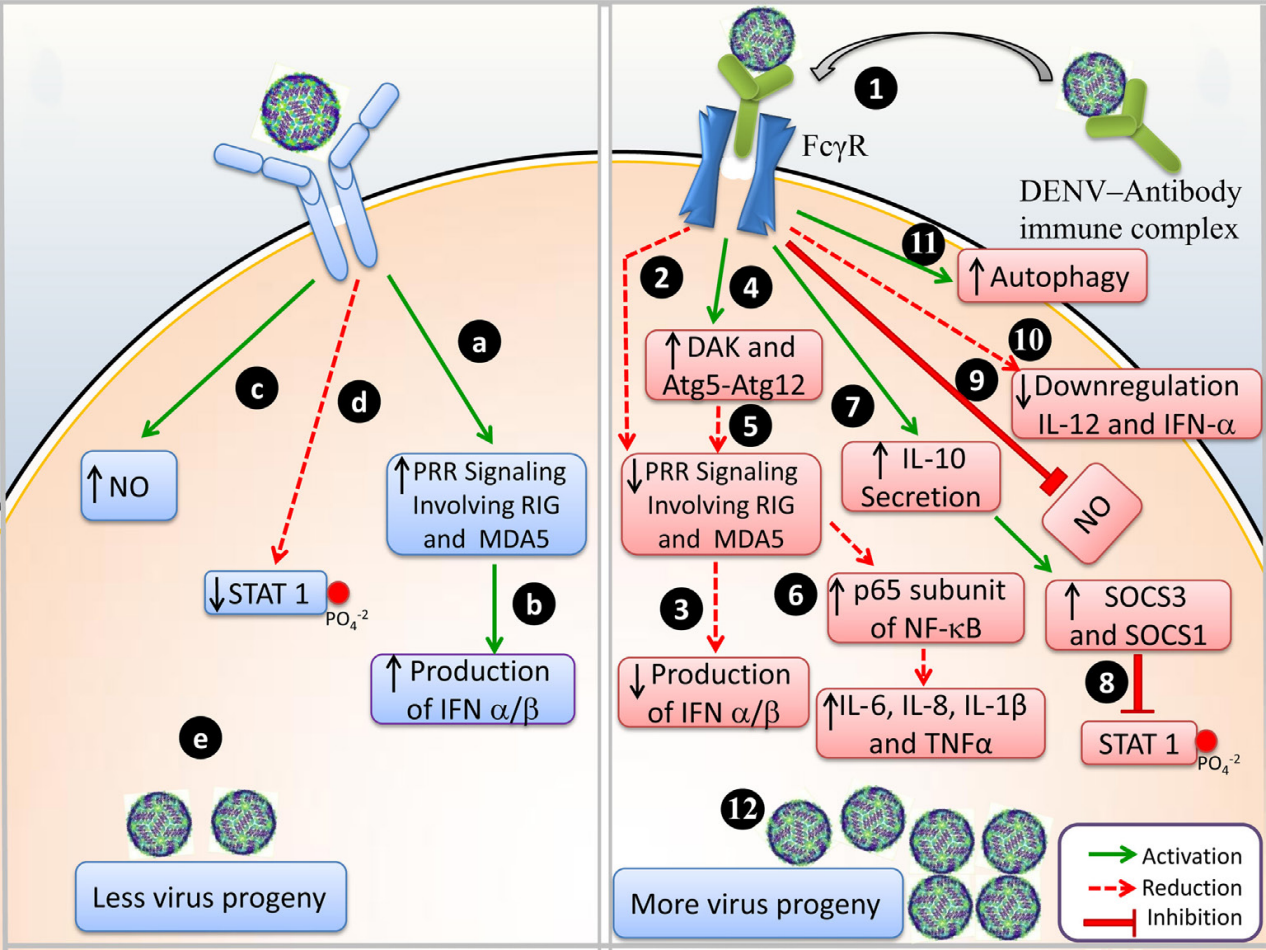
Events of immune response during Dengue virus (DENV) infection and DENV-antibody-dependent enhancement (ADE) infection. **(A)** DENV infection: (a) upon viral entry, pattern recognizing receptor (PRR) signaling activates RIG-1 and MDA-5, (b) IFN-α/β are produced, (c) NO production is upregulated, (d) phosphorylation of STAT1 is reduced, and (e) less viral progeny is produced. **(B)** DENV-ADE infection: (1) virus-antibody immune complex binds to FcγR and is trafficked inside the cell; (2) PRR signaling is not involved and RIG-1 and MDA-5 are suppressed; (3) IFN-α/β production is inhibited, (4) negative regulation of DAK and Atg5–Atg12; (5) disrupts the RIG-I/MDA-5 (PRRs) signaling cascade; (6) enhanced expression of p65 of NF-κB and degradation of I κB resulted in elevated levels of IL-6, IL-8, IL-1β, and TNF-α (pro-inflammatory cytokines); (7) IL-10 secretion enhances SOCS-3 and SOCS-1; (8) in turn attenuates STAT1 phosphorylation; (9) downregulation of IL-12 and IFN-γ; (10) NO production is suppressed; (11) autophagy is increased; and (12) more viral progeny is produced.

Detailed molecular events during DENV infection and DENV-ADE infection are illustrated in Figure 1.

## Effects of Pre-Existing DENV Antibodies

As noted above, ADE is well established in DENV but still contentious in ZIKV infections. In a few experiments, DENV antibodies were found to mediate ADE in ZIKV infection, whereas in few protections from DENV antibodies have been observed. In one study, 119 mAbs were isolated from four ZIKV-infected patients; out of these, two had no history of DENV infection (ZIKV^+^DENV^−^) and two patients were determined DENV positive by serology (ZIKV^+^DENV^+^). These 119 mAbs were evaluated for DENV/ZIKV neutralization and compared side by side with mAbs isolated previously (103) from patients infected with DENV alone (DENV^+^ZIKV^−^). Out of the 119 mAbs, 41 were specific for the NS1 protein (10). The E proteins of ZIKV and DENV have three domains: EDI, involved in a structural change of the virus required for viral entry; EDII, the fusion loop; and EDIII, which is responsible for cellular binding (104). Interestingly, in the above experiment, out of 119 mAbs, mAbs against the EI and EII domains of ZIKV were found to be highly reactive to E protein of all DENV serotypes. This indicates that mAbs to the EI and EII domains are cross-reactive between ZIKV and DENV, where mAbs to EDIII are DENV or ZIKV specific. Thus, heterologous antibodies raised against EDI/II appear to be responsible for ADE (10). The notion is recently confirmed in a study into which DNA immunization to raise antibodies against E protein domain I/II was shown to enhance ZIKV infection, but the same was not observed with DNA immunization to the E protein domain III (105).

Several mAbs against both DENV and ZIKV have been isolated from immunized animal models, as well as from human convalescent serum. The panel of mAbs against both DENV and ZIKV, showing various features like cross-neutralization among flaviviruses, enhancement or inhibition of ADE in *in vitro* and *in vivo* models, and/or specificity for DENV or ZIKV, are presented in Tables 1 and 2. This information may be useful in selecting mAbs for various purposes such as identification of epitopes that do not elicit ADE-causing antibodies. mAbs that are broadly neutralizing but cause ADE, such 2A10G6, which was generated against the highly conserved flavivirus fusion loop peptide, may be engineered for safer prophylactic usage.

**Table 1:** Various monoclonal antibodies (mAbs) developed against DENV, capable of broader neutralization; inhibition/enhancement of antibody-dependent enhancement (ADE).

Species from which mAb is derived	Name of mAb	Domain of flavivirus involved in mAb binding	Features of mAbs	Reference
Mouse	9F12	Domain III of E glycoprotein	Neutralize all the four DENV and WNV	Rajamanonmani et al. (106)

	E60	Fusion loop peptide of domain II	Broadly neutralize other flaviviruses	Crill and Chang (107)
			No binding with FcγR	Tao and Morrison (108)
			No activation of C1q	

	4G2	Domain II of E glycoprotein	Broadly neutralize other flaviviruses	Charles and Christofferson (109)
			Cause ADE of Zika in THP-1 cells	
			Enhance ZIKV infection in K562 cell line	

Female BALB/c mice	2A10G6	Highly conserved flavivirus fusion loop peptide (98DRXW101 motif)	Broad cross-reactivity against DENV 1–4, YFV, WNV, JEV, and TBEV	Deng et al. (110)

Human	1C19	Domain II of E glycoprotein	Inhibits fusion loop (FL) antibodies and enhance ADE	Smith et al. (111)

	DV82-LALA mAb	Envelope Dimer Epitope I/II	Partial inhibition of ADE	Stettler et al. (10)

	33.3A06	–	Exhibit minimum ADE and strongly neutralize ZIKV	Priyamvada et al. (9)

	C8	Envelope Dimer Epitope 1	Form and stabilize DENV envelope dimers	Rouvinski et al. (112)
	A11	Envelope Dimer Epitope 2		

	C10	Envelope Dimer Epitope 1	High therapeutic potential (require only two doses of 10 µg to protect mice from lethal ZIKV infection)	Swanstrom et al. (113)
		Virus surface E glycoprotein––at pH 6.5	High therapeutic potential (one of the most potent mAb against ZIKV)	Zhang et al. (114)
		E glycoprotein raft structure––at pH 5.0	Prevent structural rearrangement of the E proteins––a vital step for infection.	

	1.6D and D11C	FL-specific antibodies	Cross-reactive against ZIKV in Rhesus macaque LLC-MK2 kidney epithelial cells	Costin et al. (115)
			Unable to neutralize ZIKV	Paul et al. (14)

**Table 2:** Various monoclonal antibodies (mAbs) developed against ZIKV, capable of broader neutralization; inhibition/enhancement of antibody-dependent enhancement (ADE).

Species from which mAb developed	Name of mAb	Domain of flavivirus involved in mAb binding	Special notes	Reference
BALB/c mice	D11	NS1	ZIKV specific and negligible cross-reactivity with other flaviviruses	https://thenativeantigencompany.com/product/mouse-anti-zika-virus-ns1-antibody-d11/(116)

Mice	ZV-67	lateral ridge	Neutralize African, Asian, and American strainsPassively transferred antibody protects mice from infection	Zhao et al. (117)
	ZV-54			
	ZV-48	DIII (C–C′ loop)		
	ZV-64			
	ZV-2	DIII (ABDE sheet)	Poor inhibition of ZIKV infection	
	ZV-13	DI-II	Support ADE of DENV	

Human	P1F12	A unique unidentified conformational epitope	Specific for ZIKV and did not cross-react with DENV	Magnani et al. (118)

	Z23 and Z3L1	To tertiary epitopes in envelope protein	No cross-reactivity with DENV1–DENV4	Wang et al. (119)
			Potent ZIKV-specific neutralization *in vitro*	

	ZikV-117	DII dimer-dimer interface	Neutralize African, Asian, and American strains	Stettler et al.; Zhao et al. (10, 117)
			Prophylactic use	

	ZK2B10 and ZK7C3	E glycoprotein	IC50 of 0.04 and 0.11 µg/mL, respectively, for each mAb and potently neutralizing mAb	Yu et al. (120)
			No detectable cross-reactivity with DENV1 and DENV2	

	ZKA190	DIII	Highly potent in ZIKV neutralization	Stettler et al. (10)
			No ADE in K562 cells (>1-µg/mL concentration)	
	ZKA230	Neutralizing-non-E binding	Potentially neutralize ZIKV	
			Cause ADE at higher concentrations in K562 cells	
	ZKA64	DIII	Potentially neutralize ZIKV (IC50 values of 93 ng/mL)	
			No ADE in ZIKV infections in K562 cells if more than 1-µg/mL concentration is used	
	ZKA64-LALA	DIII	Complete blocking of ADE effect	
	ZKA3	Envelope Dimer Epitope I/II	Partial neutralization of ZIKV infectivity	
	ZKA78		LALA version of antibody inhibit ADE	
	ZKA185	Envelope Dimer Epitope region (Neutralizing Non-Binder)	Does not react with recombinant envelope protein (E-protein) or EDIII	

	ZIKV-116	E, DIII, and E-FLM.	Neutralize diverse ZIKV strains	Sapparapu et al. (121)
	ZIKV-19	E and E-FLM	Weakly neutralize ZIKV infection	

	ZK2B10 and ZK7C3	E glycoprotein	Strongly neutralize ZIKV, DENV1, and DENV2	Yu et al. (120)
			Therapeutic potential due to 100% protection of mice from lethal ZIKV challenge	
	ZV67	A strand, the BC loop, the DE loop, and the FG loop	Only ZIKV neutralizing	
	ZK8-4	DI/DII	Poorly neutralizing, derived from a plasmablast B cell on day 4	
			Non-protective in mice	

## DENV Antibodies in the Enhancement of ZIKV

The mAb 4G2 enhance the amplitude of ZIKV infection 60-fold in culture supernatants and 248-fold in THP-1 cell pellets in comparison with their respective controls (108). The mAb 9F12, raised against the DENV2 domain III of E protein, has the ability to neutralize all four DENV serotypes, as well as WNV, even though, generally, the mAbs against this region are highly specific and do not participate in cross-neutralization (105). Adsorption and fusion analyses indicated that 9F12 plays a role during the early steps of virus entry. The single-chain variable fragment (ScFv) of 9F12 showed similar binding ability to that of the parent antibody, and thus has greater potential to be humanized and used for therapeutic and prophylactic purposes (106) (Table 1). In contrast to results of an experiment by Paul et al. (14), 1.6D and D11C were found to be non-neutralizing and to enhance ZIKV infection in FcR-bearing K562 cells by ~140-fold and ~275-fold, respectively.

Dejnirattisai et al. (15) showed that pre-incubation of anti-DENV mAbs with ZIKV caused an increase in ZIKV titers in U937 monocytes, which, without ADE, are relatively resistant to DENV/ZIKV infection. Thus, this study showed that ZIKV infection of U937 cells was enhanced by ADE.

Recently, a panel of 54 murine DENV and WNV mAbs was explored to determine their effects against ZIKV in *in vitro* studies. Eight out of 54 antibodies (two WNV-induced and six DENV-induced mAbs) cross-reacted with ZIKV. Out of the eight cross-reactive mAbs, two WNV-induced and one DENV-induced mAb (4G2) enhanced ZIKV infection in the human immortalized myelogenous leukemia line K562. All the enhancing antibodies belonged to isotype IgG2a (7). Similar to mAb 4G2, WNV-E18 (a WNV-elicited mAb) also bound to the DII fusion loop of the E protein, whereas WNV-E24 bound to the DIII lateral ridge of the same protein (7, 122–124). The mutated polyvalent mAb E60-hIgG1-N297Q abolished Fc-receptor binding, neutralized DENV, and prevented death in mice (125). This information regarding broadly neutralizing antibodies and their ability to cause inhibition/enhancement of ADE might be helpful in designing a vaccine that is effective against both DENV and ZIKV (Table 2).

### ADE of DENV/ZIKV in the Presence of DENV Antibodies in *In Vitro* Models

There are numerous *in vitro* studies that have supported the phenomenon of ADE. Serologically naïve monkeys were inoculated with DENV serotypes 1–4, and pre- and post-infection lymphocytes from these animals were infected with different DENV serotypes. In PBLs from uninfected monkeys, little virus replication occurred, whereas in PBLs from immune monkeys, there was a significant increase in DENV1, DENV2, and DENV4 titers (126). In BHK-21 cells that stably expressed FcγRIIA, ADE was demonstrated using both monoclonal antibody (mAb) 4G2, as well as DENV antibody-positive human serum (127). Similar research group has demonstrated ADE in DENV2-infected BHK cells using 6B6C, 3H12 DENV mAbs, and a virus dose of as little as 5 plaque-forming units (PFUs) (128). Anti-DENV human mAbs 1.6D and D11C directed against the fusion loop of the virus neutralize all four DENV serotypes. These mAbs cross-react with ZIKV but do not neutralize ZIKV, and they mediate ZIKV ADE *in vitro* in K562 cells approximately 140-fold and 275-fold for MAbs 1.6D and D11C, respectively (14). Preexisting immunity to DENV has been shown to enhance ZIKV titers in primary human macrophages, as well as in a human macrophage-derived cell line with an altered pro-inflammatory cytokine profile (129). ZIKV comprises one serotype with two lineages (Asian and African). Sera raised against each of these lineages are capable of neutralizing homologous or heterologous ZIKV lineages (130). Results of DENV antibody-mediated enhancement of ZIKV infection (15) and ZIKV infection-mediated enhancement of DENV infection have been documented (18, 131, 132). Human mAbs that recognize the viral envelope protein were isolated from DENV-infected patients and found to neutralize all four DENV serotypes. These mAbs resulted in DENV2 ADE in infected THP-1 cells. The F(ab′)2 fragment of these mAbs was able to neutralize all viruses in all four serotypes, similar to the parent mAb; however, no ADE activity was observed. Therefore, these mAbs are potential therapeutic candidates (133).

### ADE of DENV/ZIKV in the Presence of DENV Antibodies in *In Vivo* Models

Although various *in vitro* studies have demonstrated ADE of DENV/ZIKV in the presence of DENV antibodies, the same is not true for *in vivo* studies. DENV antibody mediation of ADE in DENV infection *in vivo* has been reported by several groups of researchers, but the same is not true for ZIKV. This indicates that the serotype of DENV responsible for secondary infection determines the severity of disease. In humans, immunity to one DENV serotype can cause more severe disease in secondary infection with dissimilar DENV serotypes (134). The cases of DHF/DSS, a severe form of dengue, were more prevalent in patients with primary DENV infections born to DENV-immune mothers and in those with secondary infections with DENV serotypes other than that of the first infection (135).

In addition, in cases of secondary DENV infections, the frequency of DHF/DSS was approximately 15- to 80-fold higher (34). In a study of 13 infants from Bangkok with primary DENV2 infections (age less than 12 months), all showed DHF/DSS when maternal DENV-2 neutralizing antibodies declined to an approximately 1:10 titer. Mothers of these infants each had a history of one or more DENV infections, and, as maternal IgG antibodies were catabolized and diluted, DENV2 titers were enhanced in infants (24). In an investigation involving 65 patients, those who reported prior natural DENV infections did not exhibit higher ZIKV titers upon subsequent ZIKV infection, and no ADE of ZIKV was observed in cases that were pre-exposed to DENV (17). In addition, results of a screening study of 405 individuals in Brazil and Mexico revealed that ZIKV neutralization titers were higher in individuals previously exposed to DENV1 than in those who were not previously exposed. An assessment of plasma from 168 children with acute DENV infection who attended two hospitals in Thailand indicated that disease severity was associated with higher viremias and secondary DENV infection; DENV2 was associated with more severe disease during secondary infection than in secondary infections with the other DENV serotypes (134).

Many studies have been conducted to evaluate DENV antibody-mediated ADE in ZIKV infections. In mice receiving DENV-positive convalescent serum, ZIKV titers were enhanced by more than 10-fold on day 3 and viremias were sustained for a longer time, in comparison with mice receiving control plasma or phosphate-buffered saline (136). In contrast to the results of experiments in mice, in two cohorts of rhesus macaques infected with ZIKV, those pre-infected with DENV did not exhibit more severe Zika symptoms than those that were not. In fact, the number of days of ZIKV viremia was reduced in comparison to the number in naive macaques (13). During secondary infections with DENV, CD8 + T cells target epitopes of the NS gene (137, 138). Recently, Wen et al. (139) compared the role of humoral immunity vs. CD8 + T cells in protection against ZIKV infection in DENV-immune mice by depletion and adoptive transfer of T cells. Protection was found to be mediated by CD8 + T cells. The results of this study were consistent with the results of murine studies, in which DENV-specific CD8 + T cells conferred protection against heterogenous DENV serotypes and ADE (140, 141).

Dengue virus ADE in ZIKV infections may be dependent on several factors (142). For example, the DENV serotype of a prior infection may affect disease severity as in the case of DENV infections, primary infection with DENV1 and a secondary infection with DENV2 or DENV3 can result in greater disease severity in the secondary DENV2 infection (143); the time interval between the DENV and ZIKV infections as with DENV infection, sequential infections within a span of less than 2 years can blunt the severity of disease in the secondary infection, whereas a wider time interval (2–20) years might result in a severe outcome (144); and number of previous DENV infections may affect enhancement for dengue, pre-exposure to two or more than two DENV infections can result in protection (145). It is also worth mentioning that various non-human primates and breeds of mice that are susceptible for DENV and ZIKV infection are available for research; however, DENV infection in these models does not recapitulate the features of human disease; and virus titers are consistently 1,000-fold less than titers found in humans (146). The same causes may affect the results of ADE as well. Therefore, prior to inferring any conclusions, the results must be analyzed in depth, and many species must be evaluated.

## Protection Against ZIKV Infection by DENV Antibodies

Both ZIKV and DENV are flaviviruses. They show approximately 60% sequence identity, so broadly neutralizing epitopes of DENV may prevent ZIKV infection. However, type-specific mAbs such as 1F4 (DENV1 neutralizing), 2D22 (DENV2 neutralizing), 5J7 (DENV3 neutralizing), and 5H2 (DENV4 neutralizing) did not neutralize ZIKV. In addition, antibodies that are moderately cross-reactive to one DENV serotype (e.g., 4G2, 1N5, and 1M7) failed to neutralize ZIKV (147). After screening a panel of 30 cross-neutralizing human DENV mAbs, 1C19 was identified as reacting to a BC loop of the conserved domain II of the E protein. The same antibody also competes with the low-potency fusion loop (FL) antibodies, which commonly precipitates ADE (111).

The mAbs generated from memory B cells (MBCs) of ZIKV-infected patients showed various levels of binding to DENV subtypes. mAbs such as ZIKV-117 exhibited binding specificity and did not react with DENV1, DENV2, DENV3, DENV4, or purified WNV E protein, in contrast, ZIKV-116 bind to DENV1-, DENV2-, and DENV4. ZIKV-117, evaluated in wild-type male C57BL/6 mice with a single dose on day 1 (6.7-mg/kg dose) or day 5 (16.7-mg/kg dose) after virus inoculation, protected the animals against infection (121). Prophylaxis or post-exposure therapy is useful in treating pregnant mice and reducing virus titer in the mother, the placental and fetal tissues. Two mAbs (Z23 and Z3L1) also exhibited ZIKV-specific neutralization *in vitro*, with no cross-reactivity to either of the DENV serotypes.

Most of the antibodies were found to target the DENV fusion-loop epitope (FLE) of the E protein, whereas only a few targeted a site, termed as envelope dimer epitope (EDE), between two subunits of the E protein in the dimer. A structural analysis of the EDE of DENV was recently conducted by Barba-Spaeth et al. (6). Two types of EDE have been identified: EDE1, which shows improved binding capacity in the absence of glycan, and EDE2, which shows improved binding capacity in the presence of glycan (6). The mAbs to the E dimer epitope 1 (EDE1) are cross-reactive, neutralize ZIKV in cell culture, and protect against infection in a murine model. The mAbs EDE1 C8 and EDE1 C10 neutralized both a 2013 French Polynesian strain of ZIKV (H/PF/2013) and a strain from the Americas in 2015 (PRVABC59). Only two 10-µg doses of the mAb EDE1 C10 were required to protect mice from ZIKV infection (113). Hence, mAbs to EDE (EDE1 C10) offer protection that is superior to that of mAbs to FL (2A10G6), in terms of the quantity required to neutralize ZIKV infection and provide protection in an animal model. In addition, mAbs to EDE were weaker enhancers of ADE compared with mAbs against the fusion loop. The mAbs to EDE outperformed, showing 90% neutralization (112).

The identification of an epitope that is functionally conserved between ZIKV and DENV may help in designing a vaccine effective against both DENV and ZIKV infection (148, 149). Kam et al. (150) screened 23 human DENV mAbs for cross-reactivity with ZIKV in Vero E6 cells. The majority of the mAbs exhibited binding to ZIKV particles, with a few showing the capacity to neutralize ZIKV. The mAb SIgN-3C showed the capacity to rescue non-pregnant mice from virus-induced morbidity and mortality, whereas, in pregnant mice, it reduced viremias in placenta and fetal organs. SIgN-3C induced ADE, but when Leu-to-Ala mutations were introduced into the Fc region of this antibody, the mutated mAb (LALA-SIgN-3C) exhibited similar neutralizing efficiency as wild-type SIgN-3C but did not induce ADE. The mAb 33.3A06, which is a plasma blast-derived mAb from DENV-infected patients, potently neutralized ZIKV and exhibited minimal ADE (9).

## Antibody Engineering to Combat ADE

Because mosquitoes are the carriers for both of these viruses, concurrent ZIKV and DENV infections cannot be excluded. The Fc region of an antibody determines its half-life in serum, as well as its effector functions, including complement-dependent cytotoxicity, antibody-dependent cellular cytotoxicity, and antibody-dependent cell phagocytosis. The Fc region of an mAb may be altered to enhance its pharmacological efficacy. Depending on the purpose, the effector function may be enhanced or decreased. For example, to treat tumors, an enhanced effector function is required, whereas antibody targets present on immune cells require a reduced effector function. Binding of antibodies to FcγRs or C1q is dependent on amino-acid residues present in the hinge region and CH2 domain. Mutations that inhibit the interaction of antibody with FcγRs may be useful in preventing ADE. During ADE in DENV-infected patients, IgGs with an affinity to FcγRIIIA are produced. Afucosylated antibodies were elevated in the patients suffering from severe disease associated with secondary DENV infections. Such afucosylated antibodies are non-neutralizing and activate Fc receptors. Thus, vaccine or therapeutic agents that inhibit afucosylated IgG1 can prevent ADE associated with DENV/ZIKV (151).

IgY is the primary immunoglobulin isotype in oviparous animals that is functional equivalent to mammalian IgG, but the use of avian-derived IgY has additional advantage of dissimilar genetic background and phylogenetic distance from mammals. Also, IgY is unable to bind mammalian FcγR (152) or other Fc-binding receptors, that reduce its ability to evoke an inflammatory response in humans (153). In the experiment of Fink et al. (154), goose-derived, purified anti-DENV2 IgY neutralized DENV2 both in *in vitro* and in AG129 mouse model and prevented ADE in *in vitro*. The goose IgY protected mice from lethal DENV challenge, by binding to the epitopes that were not previously identified (154). Thus, goose IgY is able to prevent not only DENV infection but also ADE of DENV and the same may be true for ZIKV also.

## Mutations in the Fc Region

The Fc region of an engineered antibody must be manipulated in such a way that FcγR and C1q interactions and immune effector functions are reduced in comparison with those of native human IgG1. For this purpose, various mutations have been introduced into the CH2 domain of human IgG1, and their effects on complement-dependent toxicity and ADC have been evaluated. Inhibition of the Fc N-linked aglycosylation site (155), the LALA double mutation (Leu234Ala, together with Leu235Ala) in the Fc region of the antibody, a leucine to glutamic acid substitution at position 235 of the IgG1 Fc (156), and an N297Q substitution (125) has been observed to prevent ADE in DENV. Two variants, P329A and P329G, were assessed for their binding affinity to the receptor. P329G was less reactive with that of P329A, but both showed reduced interactions with their receptors.

Aglycosylation (N297D) or the introduction of the LALA mutation has been shown to reduce the affinity between Fc and FcγR. The ability to bind with FcγRI was found to be completely abolished in a human IgG1-P329G-LALA mutant. In a C1q binding assay, LALA, N297D, and P329G Fc mutations were silent, showing no detectable binding ability. For human IgG1, the P329G mutation was found to have reduced binding affinity to many FcγRs, including FcγRI, FcγRIIa, FcγIIb, and FcγIIIa (156). Engineered human antibodies with IgG1 alterations in residues at positions 233–236 in the Fc of IgG2 and at positions 327, 330, and 331 in IgG4 markedly decreased complement-dependent and antibody-dependent toxicity (157, 158). Idusogie et al. (159) reported that an alanine substitution in human IgG1 at positions D270, K322, P329, or P331, largely hampered antibody binding with C1q and complement fixation. A broadly neutralizing human DENV mAb (D23-1G7C2-IgG1), obtained from a DENV-infected patient and developed against the envelope protein, was shown have an ADE effect.

To reduce ADE, the Fc region of D23-1G7C2-IgG1 was modified to generate antibodies of each of the IgG subclasses (IgG2–4). N297A was also introduced to reduce the affinity of these antibodies for Fcγ receptors. These changes in the Fc region resulted in reduced ADE activity in FcγRI and FcγRII-bearing THP-1 cells by the IgG2 or IgG4 subclasses. In contrast, ADE increased after it was swapped for IgG2 in FcγRII only-bearing K562 cells. This information should be exploited to obtain insight regarding antibody engineering to combat ADE against ZIKV (160). Valuable information may be obtained regarding the use of recombinant antibodies as therapeutics.

## Bispecific Antibodies

A bispecific antibody (DVD-1A1D-2A10), with anti-DENV mAb 1A1D-2 (1A1D) specific to E-DIII, which inhibits attachment of DENV to a cell, and mAb 2A10, which binds to E-DII and prevents endosomal fusion of virus, show more potent neutralization than the individual antibodies. Recently, nine amino acids (positions 231–239) in the Fc domain of the DVD-1A1D-2A10 antibody were mutated at the N terminus of the Fc domain. The resulting antibodies showed a high affinity to DENV and neutralized all DENV serotypes without inducing ADE (161). Thus, similar strategies could be evaluated for the treatment of ZIKV infections.

A bispecific antibody named Fabs-in-tandem (FIT)-1, comprised on two mAbs; ZKA190 and ZKA185, which binds to DIII and DII of E protein, respectively, has been constructed. The FIT-1 has showed high *in vitro* and *in vivo* neutralization abilities along with capability to prevent viral escape mutant formation; therefore, it is having immunotherapeutic potential (162).

## Anti-Idiotypic Antibodies

An anti-idiotypic antibody is an antibody against the antigen binding site [complementarity determining region (CDR)] of another antibody. In the case of DENV, enhancing antibodies are specific mainly to the structural pre-membrane protein (prM). Anti-idiotypic antibodies (prM-AIDs) targeting prM mAb are generated by immunizing BALB/c mice. These polyclonal prM-AIDs greatly reduced ADE in K562 cells. In an AG6 mice model, the administration of prM-AIDs reduced ADE and decreased levels of IL-10 and ALT. This suggests that instead of using prM mAb, using prM-AIDs may be a better choice to prevent ADE during DENV infection (119). The human mAb HM14c10, obtained from a DENV patient who recovered, showed strong neutralization of DENV1 in an AG129 mouse model.

## Fab Antibodies

The Fab fragment is a part of antibody that is capable of binding with antigen, but is monovalent and lacks the Fc region. The Fab fragment of therapeutic mAb ZIKV-117 has been shown to be effective in preventing fetal infection and death in mice model. Structural analysis revealed that ZIKV-117 Fab antibodies cross-link with the monomeric units of surface E glycoprotein dimers and prevent reorganization of E protein monomers into fusogenic trimers in endosomal acidic environment (163).

The Humanyx Fab phage library was screened for the antigen HM14c10, and an E1 anti-idiotypic antibody against HM14c10 was found to potently neutralize DENV. The same could be explored to develop therapeutics (164). E1 was able to detect HM14c10- and HM14c10-like antibodies in the sera of patients who recovered from DENV infection (165), which suggested that it is a common idiotype and could be further tailored to obtain the desired results, including broader neutralization capability and diminished ADE.

**Figure 2:**
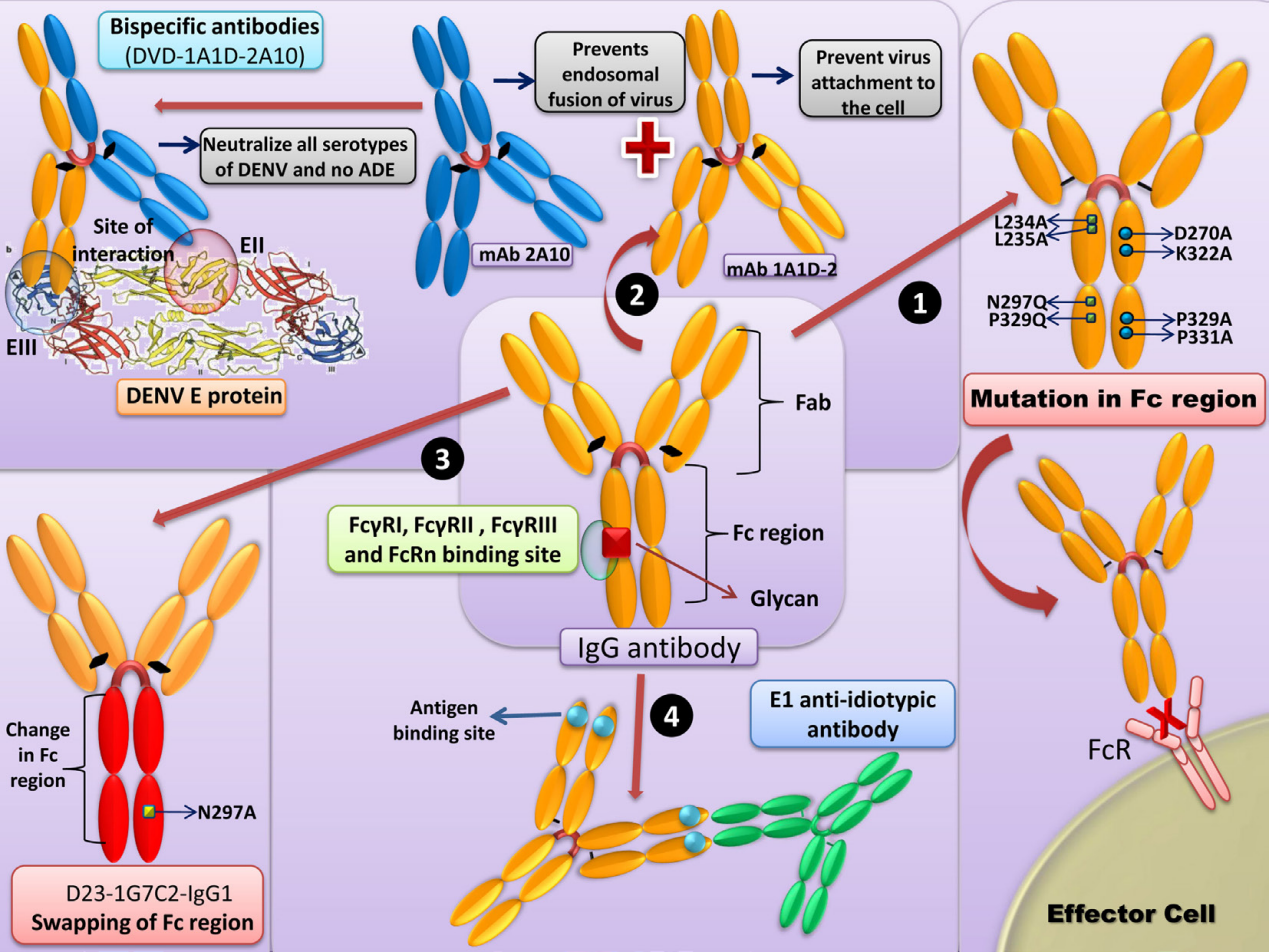
Strategies to counter Dengue virus (DENV)-antibody-dependent enhancement (ADE)/Zika virus (ZIKV). (1) *Mutations in the Fc region*: removal of native Fc N-linked glycosylation site and the “LALA” double mutation (Leu234Ala together with Leu235Ala) in the Fc region of antibody prevents binding to FcγR. (2) *Bispecific antibodies*: bispecific antibody (DVD-1A1D-2A10) with anti-DENV monoclonal antibody (mAb) 1A1D-2 (1A1D) specific to the E-DIII, check the virus attachment to the cell, and mAb 2A10, will bind to the E-DII and further prevents the endosomal fusion of the virus. This neutralizing antibody neutralizes all the DENV serotypes without inducing ADE. Similar strategies can be employed for the ZIKV. (3) *Swapping of Fc region*: the Fc region of D23-1G7C2-IgG1 can be changed to generate antibodies with each of IgG subclasses (IgG2–4). The swapping of the Fc region might result in reduced ADE activity in FcγRI and FcγRII-bearing THP-1 cells in the case of IgG2 or IgG4 subclasses. (4) *Anti-idiotypic antibodies*: anti-idiotypic antibodies (prM-AIDs) targeted to prM mAb largely reduces the symptoms of ADE.

The salient strategies to counter DENV/ZIKV ADE are summarized in Figure 2.

## Conclusion and Future Prospects

Zika virus and DENV are both vector-borne members of the *Flaviviridae* family. There is high similarity among the members of this family at the nucleotide and amino-acid levels, and this can lead to the development of cross-protective and cross-reactive antibodies. In the same geographical area, ZIKV and DENV infections may lead to generation of antibodies that might be either neutralizing or non-neutralizing. Non-neutralizing cross-reactive antibodies may lead to ADE, which is a well-known phenomenon in DENV but has not been confirmed in ZIKV infections. As most countries with confirmed ZIKV cases are also endemic for DENV, there is higher probability that ZIKV intensity may be amplified, owing to preexisting DENV cross-reactive antibodies. At present, there is evidence for the existence of *in vitro* ZIKV ADE in the presence of preexisting DENV antibodies, but in the rhesus macaque model of ADE, a reduction in viremia was observed, which indicates differences between *in vitro* and *in vivo* models. The underlying reason may be the involvement of CD8 + T-cell response in protection. These differences between *in vitro* and *in vivo* results must be understood for the successful design of a single vaccine that will provide immunity against both DENV and ZIKV. Above, we also noted that antibodies generated against EDI/II were mainly cross-reactive and might contribute to ADE of ZIKV; hence, further study is required to identify the specific region responsible for this cross-reaction. Similarly, studies should also be designed to determine whether antibodies against all serotypes of DENV cause a similar intensity of ADE in ZIKV infections.

Apart that various other factors, including the DENV serotype responsible for primary infection, the time interval between DENV and ZIKV infections and parity of DENV serotypes prior to infection with ZIKV must be taken into account to confirm ADE of ZIKV due to preexisting DENV antibodies. For this purpose, case control studies in different DENV-ZIKV endemic areas in people belonging to similar age, geographic area, ethnicity, sex, etc. Not much is known about the reciprocal response of anti-ZIKV antibodies to the enhancement of DENV infections; however, some reports have confirmed ADE of DENV in the presence of preexisting ZIKV antibodies. Studies are warranted to determine whether ADE of ZIKV can occur in the presence of antibodies to other flaviviruses. Further insights into the molecular aspects of DENV-ADE of ZIKV will assist the development of better therapeutic approaches and might also aid in the development of a pan-DENV/ZIKV vaccine. Until ADE of ZIKV infections by preexisting DENV antibodies is established, information regarding engineered antibodies and avian egg antibody will be helpful in the design of therapies with improved efficacy and safety against DENV and ZIKV infections.

## Author Contributions

All the authors substantially contributed to the conception, design, analysis and interpretation of data, checking and approving final version of manuscript, and agreed to be accountable for its contents. RK and AM initiated this review compilation. KD and YM reviewed and edited. RK designed tables. AM and KK designed the figures. KK and RT updated few critical aspects. RS reviewed virological and immunological aspects. WC reviewed the antibody engineering section. KD finally updated, edited, and overviewed.

## Conflict of Interest Statement

All authors declare that there exist no commercial or financial relationships that could in any way lead to a potential conflict of interest. The reviewer EV, LL and handling Editor declared their shared affiliation.

## References

[1] Singh RK Dhama K Malik YS Ramakrishnan MA Karthik K Tiwari R Zika virus––emergence, evolution, pathology, diagnosis, and control: current global scenario and future perspectives––a comprehensive review. Vet Q (2016) 36(3):150–75. 10.1080/01652176.2016.1188333 27158761

[2] Mayer SV Tesha RB Vasilakis N. The emergence of arthropod-borne viral diseases: a global prospective on dengue, chikungunya and Zika fevers. Acta Trop (2017) 166:155–63. 10.1016/j.actatropica.2016.11.020 27876643 PMC5203945

[3] Dhama K Karthik K Tiwari R Khandia R Munjal A Chakraborty S Zika virus/Zika fever: a comprehensive update. J Exp Biol Agric Sci (2018) 6(1):1–31. 10.18006/2018.6(1)0.1.31

[4] Singh RK Dhama K Khandia R Munjal A Karthik K Tiwari R Prevention and control strategies to counter Zika virus, a special focus on intervention approaches against vector mosquitoes—current updates. Front Microbiol (2018) 9:87 10.3389/fmicb.2018.00087 29472902 PMC5809424

[5] Chong LD Single serotype for Zika. Science (2016) 353(6301):787–8. 10.1126/science.353.6301.787-b

[6] Barba-Spaeth G Dejnirattisai W Rouvinski A Vaney MC Medits I Sharma A Structural basis of potent Zika-dengue virus antibody cross-neutralization. Nature (2016) 536(7614):48–53. 10.1038/nature18938 27338953

[7] Willis E Hensley SE. Characterization of Zika virus binding and enhancement potential of a large panel of flavivirus murine monoclonal antibodies. Virology (2017) 508:1–6. 10.1016/j.virol.2017.04.031 28475924 PMC5539878

[8] Xu X Vaughan K Weiskopf D Grifoni A Diamond MS Sette A Identifying candidate targets of immune responses in Zika virus based on homology to epitopes in other flavivirus species. PLoS Curr (2016) 8. 10.1371/currents.outbreaks.9aa2e1fb61b0f632f58a098773008c4b 28018746 PMC5145810

[9] Priyamvada L Quicke KM Hudson WH Onlamoon N Sewatanon J Edupuganti S Human antibody responses after dengue virus infection are highly cross-reactive to Zika virus. Proc Natl Acad Sci U S A (2016) 113(28):7852–7. 10.1073/pnas.1607931113 27354515 PMC4948328

[10] Stettler K Beltramello M Espinosa DA Graham V Cassotta A Bianchi S Specificity, cross-reactivity, and function of antibodies elicited by Zika virus infection. Science (2016) 353(6301):823–6. 10.1126/science.aaf8505 27417494

[11] Lanciotti RS Kosoy OL Laven JJ Velez JO Lambert AJ Johnson AJ Genetic and serologic properties of Zika virus associated with an epidemic, Yap State, Micronesia, 2007. Emerg Infect Dis (2008) 14(8):1232–9. 10.3201/eid1408.080287 18680646 PMC2600394

[12] Robbiani DF Bozzacco L Keeffe JR Khouri R Olsen PC Gazumyan A Recurrent potent human neutralizing antibodies to Zika virus in Brazil and Mexico. Cell (2017) 169(4):597–609. 10.1016/j.cell.2017.04.024 28475892 PMC5492969

[13] Pantoja P Pérez-Guzmán EX Rodríguez IV White LJ González O Serrano C Zika virus pathogenesis in rhesus macaques is unaffected by pre-existing immunity to dengue virus. Nat Commun (2017) 8:15674. 10.1038/ncomms15674 28643775 PMC5490051

[14] Paul LM Carlin ER Jenkins MM Tan AL Barcellona CM Nicholson CO Dengue virus antibodies enhance Zika virus infection. Clin Transl Immunology (2016) 5(12):e117. 10.1038/cti.2016.72 28090318 PMC5192063

[15] Dejnirattisai W Supasa P Wongwiwat W Rouvinski A Barba-Spaeth G Duangchinda T Dengue virus sero-cross-reactivity drives antibody-dependent enhancement of infection with Zika virus. Nat Immunol (2016) 17(9):1102–8. 10.1038/ni.3515 27339099 PMC4994874

[16] Kalayanarooj S Nimmannitya S. Clinical presentations of dengue hemorrhagic fever in infants compared to children. J Med Assoc Thai (2003) 86(3):S673–80. 14700166

[17] Terzian ACB Schanoski AS Mota MTDO Silva RAD Estofolete CF Colombo TE Viral load and cytokine response profile does not support antibody-dependent enhancement in dengue-primed Zika-infected patients. Clin Infect Dis (2017) 65(8):1260–5. 10.1093/cid/cix558 29017246 PMC5849103

[18] George J Valiant WG Mattapallil MJ Walker M Huang YS Vanlandingham DL Prior exposure to Zika virus significantly enhances peak dengue-2 viremia in rhesus macaques. Sci Rep (2017) 7:10498. 10.1038/s41598-017-10901-1 28874759 PMC5585353

[19] Ubol S Phuklia W Kalayanarooj S Modhiran N. Mechanisms of immune evasion induced by a complex of dengue virus and preexisting enhancing antibodies. J Infect Dis (2010) 201(6):923–35. 10.1086/651018 20158392

[20] Rolph MS Zaid A Rulli NE Mahalingam S Downregulation of interferon-β in antibody-dependent enhancement of dengue viral infections of human macrophages is dependent on interleukin-6. J Infect Dis (2011) 204(3):489–91. 10.1093/infdis/jir271 21742851

[21] Zompi S Harris E. Animal models of dengue virus infection. Viruses (2012) 4(1):62–82. 10.3390/v4010062 22355452 PMC3280519

[22] Halstead SB Shotwell H Casals J Studies on the pathogenesis of dengue infection in monkeys. II. Clinical laboratory responses to heterologous infection. J Infect Dis (1973) 128(1):15–22. 10.1093/infdis/128.1.7 4198024

[23] Libraty DH Acosta LP Tallo V Segubre-Mercado E Bautista A Potts JA A prospective nested case-control study of dengue in infants: rethinking and refining the antibody-dependent enhancement dengue hemorrhagic fever model. PLoS Med (2009) 6(10):e1000171. 10.1371/journal.pmed.1000171 19859541 PMC2762316

[24] Kliks SC Nimmanitya S Nisalak A Burke DS. Evidence that maternal dengue antibodies are important in the development of dengue hemorrhagic fever in infants. Am J Trop Med Hyg (1988) 38(2):411–9. 10.4269/ajtmh.1988.38.411 3354774

[25] Hawkes RA Enhancement of the infectivity of arboviruses by specific antisera produced indomestic fowls. Aust J Exp Biol Med Sci (1964) 42:465–82. 10.1038/icb.1964.44 14202187

[26] Chaichana P Okabayashi T Puiprom O Sasayama M Sasaki T Yamashita A Low levels of antibody-dependent enhancement *in vitro* using viruses and plasma from dengue patients. PLoS One (2014) 9(3):e92173. 10.1371/journal.pone.0092173 24642752 PMC3958444

[27] Castanha PMS Nascimento EJM Braga C Cordeiro MT de Carvalho OV de Mendonça LR Dengue virus-specific antibodies enhance Brazilian Zika virus infection. J Infect Dis (2017) 215(5):781–5. 10.1093/infdis/jiw638 28039355 PMC5854042

[28] Takada A Feldmann H Ksiazek TG Kawaoka Y. Antibody-dependent enhancement of Ebola virus infection. J Virol (2003) 77(13):7539–44. 10.1128/JVI.77.13.7539-7544.2003 12805454 PMC164833

[29] Willey S Aasa-Chapman MM O’Farrell S Pellegrino P Williams I Weiss RA Extensive complement-dependent enhancement of HIV-1 by autologous non-neutralising antibodies at early stages of infection. Retrovirology (2011) 8:16. 10.1186/1742-4690-8-16 21401915 PMC3065417

[30] Kanno H Wolfinbarger JB Bloom ME. Aleutian mink disease parvovirus infection of mink macrophages and human macrophage cell line U937: demonstration of antibody-dependent enhancement of infection. J Virol (1993) 67(12):7017–24. 8230426 10.1128/jvi.67.12.7017-7024.1993 PMC238162

[31] Sauter P Hober D. Mechanisms and results of the antibody-dependent enhancement of viral infections and role in the pathogenesis of coxsackievirus B-induced diseases. Microbes Infect (2009) 11(4):443–51. 10.1016/j.micinf.2009.09.011 19399964

[32] Raabe ML Issel CJ Montelaro RC. *In vitro* antibody-dependent enhancement assays are insensitive indicators of *in vivo* vaccine enhancement of equine infectious anemia virus. Virology (1999) 259(2):416–27. 10.1006/viro.1999.9772 10388665

[33] Weiss RC Scott FW. Antibody-mediated enhancement of disease in feline infectious peritonitis: comparisons with dengue hemorrhagic fever. Comp Immune Microbiol Infect Dis (1981) 4:175–88. 10.1016/0147-9571(81)90003-5 6754243 PMC7134169

[34] Halstead SB Immune enhancement of viral infection. Prog Allergy (1982) 31:301–64. 6292921

[35] Mdurvwa EG Ogunbiyi PO Gakou HS Reddy PG. Pathogenic mechanisms of caprine arthritis-encephalitis virus. Vet Res Commun (1994) 18:483–90. 10.1007/BF01839425 7701786

[36] Yoon KJ Wu LL Zimmerman JJ Hill HT Platt KB. Antibody-dependent enhancement (ADE) of porcine reproductive and respiratory syndrome virus (PRRSV) infection in pigs. Viral Immunol (1996) 9:51–63. 10.1089/vim.1996.9.51 8733920

[37] Gomez-Villamandos JC Carrasco L Bautista MJ Quezada M Hervas J Chacón Mde L African swine fever and classical swine fever: a review of the pathogenesis. Dtsch Tierarztl Wochenschr (2003) 110:165–9. 12756959

[38] Kliks SC Halstead SB. Role of antibodies and host cells in plaque enhancement of Murray Valley encephalitis virus. J Virol (1983) 46(2):394–404. 6842678 10.1128/jvi.46.2.394-404.1983 PMC255141

[39] Halstead SB Nimmannitya S Yamarat C Russell PK Hemorrhagic fever in Thailand; recent knowledge regarding etiology. Jpn J Med Sci Biol (1967) 20:96–103. 5301574

[40] Marchette NJ Halstead SB Chow JS. Replication of dengue viruses in cultures of peripheral blood leukocytes from dengue-immune rhesus monkeys. J Infect Dis (1976) 133(3):274–82. 10.1093/infdis/133.3.274 815444

[41] Halstead SB. *In vivo* enhancement of dengue virus infection in rhesus monkeys by passively transferred antibody. J Infect Dis (1979) 140(4):527–33. 10.1093/infdis/140.4.527 117061

[42] Modhiran N Kalayanarooj S Ubol S. Subversion of innate defenses by the interplay between DENV and pre-existing enhancing antibodies: TLRs signaling collapse. PLoS Negl Trop Dis (2010) 4:e924. 10.1371/journal.pntd.0000924 21200427 PMC3006139

[43] Taylor A Foo SS Bruzzone R Dinh LV King NJ Mahalingam S. Fc receptors in antibody-dependent enhancement of viral infections. Immunol Rev (2015) 268(1):340–64. 10.1111/imr.12367 26497532 PMC7165974

[44] Flipse J Diosa-Toro MA Hoornweg TE van de Pol DPI Urcuqui-Inchima S Jolanda M Antibody-dependent enhancement of dengue virus infection in primary human macrophages; balancing higher fusion against antiviral responses. Sci Rep (2016) 6:29201. 10.1038/srep29201 27380892 PMC4933910

[45] Huang X Yue Y Li D Zhao Y Qiu L Chen J Antibody-dependent enhancement of dengue virus infection inhibits RLR-mediated type-I IFN-independent signalling through upregulation of cellular autophagy. Sci Rep (2016) 6:22303. 10.1038/srep22303 26923481 PMC4770412

[46] Ng JKW Zhang SL Tan HC Yan B Gomez JMM Tan WY First experimental *in vivo* model of enhanced dengue disease severity through maternally acquired heterotypic dengue antibodies. PLoS Pathog (2014) 10(4):e1004031. 10.1371/journal.ppat.1004031 24699622 PMC3974839

[47] MacMicking JD. Interferon-inducible effector mechanisms in cell-autonomous immunity. Nat Rev Immunol (2012) 12(5):367–82. 10.1038/nri3210 22531325 PMC4150610

[48] Lubick KJ Robertson SJ McNally KL Freedman BA Rasmussen AL Taylor RT Flavivirus antagonism of type I interferon signaling reveals prolidase as a regulator of IFNAR1 surface expression. Cell Host Microbe (2015) 18(1):61–74. 10.1016/j.chom.2015.06.007 26159719 PMC4505794

[49] Morrison J Laurent-Rolle M Maestre AM Rajsbaum R Pisanelli G Simon V Dengue virus co-opts UBR4 to degrade STAT2 and antagonize type I interferon signaling. PLoS Pathog (2013) 9(3):e1003265. 10.1371/journal.ppat.1003265 23555265 PMC3610674

[50] Grant A Ponia SS Tripathi S Balasubramaniam V Miorin L Sourisseau M Zika virus targets human STAT2 to inhibit type I interferon signaling. Cell Host Microbe (2016) 19(6):882–90. 10.1016/j.chom.2016.05.009 27212660 PMC4900918

[51] Chareonsirisuthigul T Kalayanarooj S Ubol S. Dengue virus (DENV) antibody-dependent enhancement of infection upregulates the production of anti-inflammatory cytokines, but suppresses anti-DENV free radical and pro-inflammatory cytokine production, in THP-1 cells. J Gen Virol (2007) 88(Pt 2):365–75. 10.1099/vir.0.82537-0 17251552

[52] Takhampunya R Padmanabhan R Ubol S. Antiviral action of nitric oxide on dengue virus type 2 replication. J Gen Virol (2006) 87(Pt 10):3003–11. 10.1099/vir.0.81880-0 16963759

[53] Yoneyama M Onomoto K Jogi M Akaboshi T Fujita T. Viral RNA detection by RIG-I-like receptors. Curr Opin Immunol (2015) 32:48–53. 10.1016/j.coi.2014.12.012 25594890

[54] Reikine S Nguyen JB Modis Y. Pattern recognition and signaling mechanisms of RIG-I and MDA5. Front Immunol (2014) 5:342. 10.3389/fimmu.2014.00342 25101084 PMC4107945

[55] Lin JC Lin SC Chen WY Yen YT Lai CW Tao MH Dengue viral protease interaction with NF-κB inhibitor α/β results in endothelial cell apoptosis and hemorrhage development. J Immunol (2014) 193(3):1258–67. 10.4049/jimmunol.1302675 24973451

[56] Nguyen TH Lei HY Nguyen TL Lin YS Huang KJ Le BL Dengue hemorrhagic fever in infants: a study of clinical and cytokine profiles. J Infect Dis (2004) 189:221–32. 10.1086/380762 14722886

[57] Boonnak K Dambach KM Donofrio GC Tassaneetrithep B Marovich MA. Cell type specificity and host genetic polymorphisms influence antibody-dependent enhancement of dengue virus infection. J Virol (2011) 85(4):1671–83. 10.1128/JVI.00220-10 21123382 PMC3028884

[58] Pérez AB García G Sierra B Alvarez M Vázquez S Cabrera MV IL-10 levels in dengue patients: some findings from the exceptional epidemiological conditions in Cuba. J Med Virol (2004) 73(2):230–4. 10.1002/jmv.20080 15122797

[59] Yeh WT Chen RF Wang L Liu JW Shaio MF Yang KD Implications of previous subclinical dengue infection but not virus load in dengue hemorrhagic fever. FEMS Immunol Med Microbiol (2006) 48(1):84–90. 10.1111/j.1574-695X.2006.00127.x 16965355

[60] Tang Y Kou Z Zhang F Yao X Liu S Ma J Both viremia and cytokine levels associate with the lack of severe disease in secondary dengue 1 infection among adult Chinese patients. PLoS One (2010) 5(12):e15631. 10.1371/journal.pone.0015631 21206915 PMC3012067

[61] Kudchodkar SB Levine B. Viruses and autophagy. Rev Med Virol (2009) 19(6):359–78. 10.1002/rmv.630 19750559 PMC2852112

[62] Lee YR Lei HY Liu MT Wang JR Chen SH Jiang-Shieh YF Autophagic machinery activated by dengue virus enhances virus replication. Virology (2008) 374(2):240–8. 10.1016/j.virol.2008.02.016 18353420 PMC7103294

[63] Cao B Parnell LA Diamond MS Mysorekar IU. Inhibition of autophagy limits vertical transmission of Zika virus in pregnant mice. J Exp Med (2017) 214(8):2303–13. 10.1084/jem.20170957 28694387 PMC5551583

[64] Lee YR Hu HY Kuo SH Lei HY Lin YS Yeh TM Dengue virus infection induces autophagy: an *in vivo* study. J Biomed Sci (2013) 20:65. 10.1186/1423-0127-20-65 24011333 PMC3848819

[65] Jounai N Takeshita F Kobiyama K Sawano A Miyawaki A Xin KQ The Atg5 Atg12 conjugate associates with innate antiviral immune responses. Proc Natl Acad Sci U S A (2007) 104(35):14050–10455. 10.1073/pnas.0704014104 17709747 PMC1955809

[66] McLean JE Wudzinska A Datan E Quaglino D Zakeri Z. Flavivirus NS4A-induced autophagy protects cells against death and enhances virus replication. J Biol Chem (2011) 286(25):22147–59. 10.1074/jbc.M110.192500 21511946 PMC3121359

[67] Liang Q Luo Z Zeng J Chen W Foo SS Lee SA Zika virus NS4A and NS4B proteins deregulate Akt-mTOR signaling in human fetal neural stem cells to inhibit neurogenesis and induce autophagy. Cell Stem Cell (2016) 19(5):663–71. 10.1016/j.stem.2016.07.019 27524440 PMC5144538

[68] Fang YT Wan SW Lu YT Yao JH Lin CF Hsu LJ Autophagy facilitates antibody-enhanced dengue virus infection in human pre-basophil/mast cells. PLoS One (2014) 9(10):e110655. 10.1371/journal.pone.0110655 25329914 PMC4199741

[69] Ubol S Halstead SB. How innate immune mechanisms contribute to antibody-enhanced viral infections. Clin Vaccine Immunol (2010) 17(12):1829–35. 10.1128/CVI.00316-10 20876821 PMC3008185

[70] Kliks SC Halstead SB An explanation for enhanced virus plaque formation in chick embryo cells. Nature (1980) 285:504–5. 10.1038/285504a0 7402295

[71] Ayala-Nunez NV Hoornweg TE van de Pol DP Sjollema KA Flipse J van der Schaar HM How antibodies alter the cell entry pathway of dengue virus particles in macrophages. Sci Rep (2016) 6:28768. 10.1038/srep28768 27385443 PMC4935958

[72] Kubagawa H Oka S Kubagawa Y Torii I Takayama E Kang DW The long elusive IgM Fc receptor, FcμR. J Clin Immunol (2014) 34(Suppl 1):S35–45. 10.1007/s10875-014-0022-7 24793544 PMC4160156

[73] Halstead SB Mahalingam S Marovich MA Ubol S Mosser DM. Intrinsic antibody-dependent enhancement of microbial infection in macrophages: disease regulation by immune complexes. Lancet Infect Dis (2010) 10(10):712–22. 10.1016/S1473-3099(10)70166-3 20883967 PMC3057165

[74] Lazear HM Diamond MS. Zika virus: new clinical syndromes and its emergence in the Western hemisphere. J Virol (2016) 90(10):4864–75. 10.1128/JVI.00252-16 26962217 PMC4859708

[75] Beatty PR Puerta-Guardo H Killingbeck SS Glasner DR Hopkins K Harris E. Dengue virus NS1 triggers endothelial permeability and vascular leak that is prevented by NS1 vaccination. Sci Transl Med (2015) 7(304):304ra141. 10.1126/scitranslmed.aaa3787 26355030

[76] Chung KM Nybakken GE Thompson BS Engle MJ Marri A Fremont DH Antibodies against West Nile virus nonstructural protein NS1 prevent lethal infection through Fc gamma receptor-dependent and -independent mechanisms. J Virol (2006) 80(3):1340–51. 10.1128/JVI.80.3.1340-1351.2006 16415011 PMC1346945

[77] Hunter S Indik ZK Kim MK Cauley MD Park JG Schreiber AD. Inhibition of Fc gamma receptor-mediated phagocytosis by a nonphagocytic Fc gamma receptor. Blood (1998) 91:1762–8. 9473244

[78] Tsunoda I Omura S Sato F Kusunoki S Fujita M Park AM Neuropathogenesis of Zika virus infection: potential roles of antibody-mediated pathology. Acta Med Kinki Univ (2016) 41(2):37–52. 28428682 PMC5395253

[79] Moi ML Lim CK Takasaki T Kurane I. Involvement of the Fc gamma receptor IIA cytoplasmic domain in antibody-dependent enhancement of dengue virus infection. J Gen Virol (2010) 91:103–11. 10.1099/vir.0.014829-0 19776239

[80] Mero P Zhang CY Huang ZY Kim MK Schreiber AD Grinstein S Phosphorylation-independent ubiquitylation and endocytosis of FcγRIIA. J Biol Chem (2006) 281(44):33242–9. 10.1074/jbc.M605372200 16959774

[81] Gollins SW Porterfield JS. Flavivirus infection enhancement in macrophages: an electron microscopic study of viral cellular entry. J Gen Virol (1985) 66(Pt 9):1969–82. 10.1099/0022-1317-66-9-1969 4031825

[82] Karp CL el-Safi SH Wynn TA Satti MM Kordofani AM Hashim FA In *vivo* cytokine profiles in patients with kala-azar. Marked elevation of both interleukin-10 and interferon-gamma. J Clin Invest (1993) 91(4):1644–8. 10.1172/JCI116372 8097208 PMC288142

[83] Green S Vaughn DW Kalayanarooj S Nimmannitya S Suntayakorn S Nisalak A Elevated plasma interleukin-10 levels in acute dengue correlate with disease severity. J Med Virol (1999) 59(3):329–46. 10.1002/(SICI)1096-9071(199911)59:3<329::AID-JMV12>3.0.CO;2-G 10502265

[84] Riedinger HJ van Betteraey M Probst H. Hypoxia blocks in vivo initiation of simian virus 40 replication at a stage preceding origin unwinding. J Virol (1999) 73:2243–52. 9971807 10.1128/jvi.73.3.2243-2252.1999 PMC104469

[85] Pipiya T Sauthoff H Huang YQ Chang B Cheng J Heitner S Hypoxia reduces adenoviral replication in cancer cells by downregulation of viral protein expression. Gene Ther (2005) 12:911–7. 10.1038/sj.gt.3302459 15690061

[86] Vassilaki N Kalliampakou KI Kotta-Loizou I Befani C Liakos P Simos G Low oxygen tension enhances hepatitis C virus replication. J Virol (2013) 87:2935–48. 10.1128/JVI.02534-12 23269812 PMC3571389

[87] Halstead SB O’Rourke EJ Allison AC. Dengue viruses and mononuclear phagocytes. II. Identity of blood and tissue leukocytes supporting *in vitro* infection. J Exp Med (1977) 146(1):218–29. 10.1084/jem.146.1.218 195000 PMC2180735

[88] Noisakran S Onlamoon N Songprakhon P Hsiao HM Chokephaibulkit K Perng GC. Cells in dengue virus infection *in vivo*. Adv Virol (2010) 2010:164878. 10.1155/2010/164878 22331984 PMC3276057

[89] Aye KS Charngkaew K Win N Wai KZ Moe K Punyadee N Pathologic highlights of dengue hemorrhagic fever in 13 autopsy cases from Myanmar. Hum Pathol (2014) 45:1221–33. 10.1016/j.humpath.2014.01.022 24767772

[90] Prestwood TR May MM Plummer EM Morar MM Yauch LE Shresta S. Trafficking and replication patterns reveal splenic macrophages as major targets of dengue virus in mice. J Virol (2012) 86:12138–47. 10.1128/JVI.00375-12 22933295 PMC3486461

[91] Carreau A HafnyRahbi BE Matejuk A Grillon C Kieda C. Why is the partial oxygen pressure of human tissues a crucial parameter? Small molecules and hypoxia. J Cell Mol Med (2011) 15:1239–53. 10.1111/j.1582-4934.2011.01258.x 21251211 PMC4373326

[92] Bosco MC Pierobon D Blengio F Raggi F Vanni C Gattorno M Hypoxia modulates the gene expression profile of immunoregulatory receptors in human mDCs: identification of TREM-1 as a novel hypoxic marker *in vitro* and *in vivo*. Blood (2011) 117:2625–39. 10.1182/blood-2010-06-292136 21148811

[93] Gan ES Cheong WF Chan KR Ong EZ Chai X Tan HC Hypoxia enhances antibody-dependent dengue virus infection. EMBO J (2017) 36(10):1348–63. 10.15252/embj.201695642 28320741 PMC5430213

[94] Xu H Lu A Sharp FR. Regional genome transcriptional response of adult mouse brain to hypoxia. BMC Genomics (2011) 12:499. 10.1186/1471-2164-12-499 21988864 PMC3218040

[95] Rothwell C LeBreton A Ng CY Lim JYH Liu W Vasudevan S Cholesterol biosynthesis modulation regulates dengue virus replication. Virology (2009) 389:8–19. 10.1016/j.virol.2009.03.025 19419745

[96] Kyle JL Beatty PR Harris E. Dengue virus infects macrophages and dendritic cells in a mouse model of infection. J Infect Dis (2007) 195:1808–17. 10.1086/518007 17492597

[97] Unkeless JC Shen Z Lin CW DeBeus E. Function of human Fc gamma RIIA and Fc gamma RIIIB. Semin Immunol (1995) 7(1):37–44. 10.1016/1044-5323(95)90006-3 7612894

[98] Bournazos S Ravetch JV Fcγ receptor pathways during active and passive immunization. Immunol Rev (2015) 268(1):88–103. 10.1111/imr.12343 26497515 PMC7556827

[99] Suzuki T Suzuki Y. Virus infection and lipid rafts. Biol Pharm Bull (2006) 29:1538–41. 10.1248/bpb.29.1538 16880600

[100] Cruz-Oliveira C Freire JM Conceição TM Higa LM Castanho MA Da Poian AT. Receptors and routes of dengue virus entry into the host cells. FEMS Microbiol Rev (2015) 39(2):155–70. 10.1093/femsre/fuu004 25725010

[101] Puerta-Guardo H Mosso C Medina F Liprandi F Ludert JE del Angel RM. Antibody-dependent enhancement of dengue virus infection in U937 cells requires cholesterol-rich membrane microdomains. J Gen Virol (2010) 91:394–403. 10.1099/vir.0.015420-0 19828759

[102] Baek S Kim SM Lee SA Rhim BY Eo SK Kim K. The cholesterol-binding antibiotic nystatin induces expression of macrophage inflammatory protein-1 in macrophages. Biomol Ther (Seoul) (2013) 21(1):42–8. 10.4062/biomolther.2012.082 24009857 PMC3762298

[103] Beltramello M Williams KL Simmons CP Macagno A Simonelli L Quyen NT The human immune response to dengue virus is dominated by highly cross-reactive antibodies endowed with neutralizing and enhancing activity. Cell Host Microbe (2010) 8(3):271–83. 10.1016/j.chom.2010.08.007 20833378 PMC3884547

[104] Modis Y Ogata S Clements D Harrison SC. A ligand-binding pocket in the dengue virus envelope glycoprotein. Proc Natl Acad Sci U S A (2003) 100:6986–91. 10.1073/pnas.0832193100 12759475 PMC165817

[105] Slon Campos JL Poggianella M Marchese S Mossenta M Rana J Arnoldi F DNA-immunisation with dengue virus E protein domains I/II, but not domain III, enhances Zika, West Nile and Yellow Fever virus infection. PLoS One (2017) 12(7):e0181734. 10.1371/journal.pone.0181734 28742857 PMC5526558

[106] Rajamanonmani R Nkenfou C Clancy P Yau YH Shochat SG Sukupolvi-Petty S On a mouse monoclonal antibody that neutralizes all four dengue virus serotypes. J Gen Virol (2009) 90(Pt 4):799–809. 10.1099/vir.0.006874-0 19264660 PMC2889437

[107] Crill WD Chang GJ. Localization and characterization of flavivirus envelope glycoprotein cross-reactive epitopes. J Virol (2004) 78(24):13975–86. 10.1128/JVI.78.24.13975-13986.2004 15564505 PMC533943

[108] Tao MH Morrison SL. Studies of aglycosylated chimeric mouse-human IgG. Role of carbohydrate in the structure and effector functions mediated by the human IgG constant region. J Immunol (1989) 143(8):2595–601. 2507634

[109] Charles AS Christofferson RC. Utility of a dengue-derived monoclonal antibody to enhance Zika infection *in vitro*. PLoS Curr (2016) 8. 10.1371/currents.outbreaks.4ab8bc87c945eb41cd8a49e127082620 27660733 PMC5026288

[110] Deng YQ Dai JX Ji GH Jiang T Wang HJ Yang HO A broadly flavivirus cross-neutralizing monoclonal antibody that recognizes a novel epitope within the fusion loop of E protein. PLoS One (2011) 6(1):e16059. 10.1371/journal.pone.0016059 21264311 PMC3019176

[111] Smith SA de Alwis AR Kose N Harris E Ibarra KD Kahle KM The potent and broadly neutralizing human dengue virus-specific monoclonal antibody 1C19 reveals a unique cross-reactive epitope on the bc loop of domain II of the envelope protein. MBio (2013) 4(6):e873–813. 10.1128/mBio.00873-13 24255124 PMC3870244

[112] Rouvinski A Guardado-Calvo P Barba-Spaeth G Duquerroy S Vaney MC Kikuti CM Recognition determinants of broadly neutralizing human antibodies against dengue viruses. Nature (2015) 520(7545):109–13. 10.1038/nature14130 25581790

[113] Swanstrom JA Plante JA Plante KS Young EF McGowan E Gallichotte EN Dengue virus envelope dimer epitope monoclonal antibodies isolated from dengue patients are protective against Zika virus. MBio (2016) 7(4):e1123–1116. 10.1128/mBio.01123-16 27435464 PMC4958264

[114] Zhang S Kostyuchenko VA Ng TS Lim XN Ooi JSG Lambert S Neutralization mechanism of a highly potent antibody against Zika virus. Nat Commun (2016) 7:13679. 10.1038/ncomms13679 27882950 PMC5123051

[115] Costin JM Zaitseva E Kahle KM Nicholson CO Rowe DK Graham AS Mechanistic study of broadly neutralizing human monoclonal antibodies against dengue virus that target the fusion loop. J Virol (2013) 87(1):52–66. 10.1128/JVI.02273-12 23077306 PMC3536401

[116] Available from: https://thenativeantigencompany.com/product/mouse-anti-zika-virus-ns1-antibody-d11/ (accessed December 26, 2017).

[117] Zhao H Fernandez E Dowd KA Speer SD Platt DJ Gorman MJ Structural basis of Zika virus-specific antibody protection. Cell (2016) 166(4):1016–27. 10.1016/j.cell.2016.07.020 27475895 PMC4983199

[118] Magnani DM Silveira CGT Rosen BC Ricciardi MJ Pedreño-Lopez N Gutman MJ A human inferred germline antibody binds to an immunodominant epitope and neutralizes Zika virus. PLoS Negl Trop Dis (2017) 11(6):e0005655. 10.1371/journal.pntd.0005655 28604797 PMC5481143

[119] Wang M Yang F Huang D Huang Y Zhang X Wang C Anti-idiotypic antibodies specific to prM monoantibody prevent antibody dependent enhancement of dengue virus infection. Front Cell Infect Microbiol (2017) 7:157. 10.3389/fcimb.2017.00157 28536674 PMC5422453

[120] Yu L Wang R Gao F Li M Liu J Wang J Delineating antibody recognition against Zika virus during natural infection. JCI Insight (2017) 2(12):e93042. 10.1172/jci.insight.93042 28614803 PMC5470883

[121] Sapparapu G Fernandez E Kose N Cao B Fox JM Bombardi RG Neutralizing human antibodies prevent Zika virus replication and fetal disease in mice. Nature (2016) 540:443–7. 10.1038/nature20564 27819683 PMC5583716

[122] Oliphant T Engle M Nybakken GE Doane C Johnson S Huang L Development of a humanized monoclonal antibody with therapeutic potential against West Nile virus. Nat Med (2005) 11:522–30. 10.1038/nm1240 15852016 PMC1458527

[123] Oliphant T Nybakken GE Engle M Xu Q Nelson CA Sukupolvi-Petty S Antibody recognition and neutralization determinants on domains I and II of West Nile virus envelope protein. J Virol (2006) 80:12149–59. 10.1128/JVI.01732-06 17035317 PMC1676294

[124] Stiasny K Kiermayr S Holzmann H Heinz FX. Cryptic properties of a cluster of dominant flavivirus cross-reactive antigenic sites. J Virol (2006) 80:9557–68. 10.1128/JVI.00080-06 16973559 PMC1617264

[125] Balsitis SJ Williams KL Lachica R Flores D Kyle JL Mehlhop E Lethal antibody enhancement of dengue disease in mice is prevented by Fc modification. PLoS Pathog (2010) 6(2):e1000790. 10.1371/journal.ppat.1000790 20168989 PMC2820409

[126] Halstead SB Chow JS Marchette NJ Immunological enhancement of dengue virus replication. Nat New Biol (1973) 243(122):24–6. 17319077

[127] Moi ML Lim CK Kotaki A Takasaki T Kurane I. Development of an antibody-dependent enhancement assay for dengue virus using stable BHK-21 cell lines expressing Fc gammaRIIA. J Virol Methods (2010) 163(2):205–9. 10.1016/j.jviromet.2009.09.018 19781573

[128] Moi ML Takasaki T Saijo M Kurane I Determination of antibody concentration as the main parameter in a dengue virus antibody-dependent enhancement assay using FcγR-expressing BHK cells. Arch Virol (2014) 159(1):103–16. 10.1007/s00705-013-1787-3 23900750

[129] Londono-Renteria B Troupin A Cardenas JC Hall A Perez OG Cardenas L A relevant *in vitro* human model for the study of Zika virus antibody-dependent enhancement. J Gen Virol (2017) 98(7):1702–12. 10.1099/jgv.0.000833 28691657 PMC7011765

[130] Dowd KA DeMaso CR Pelc RS Speer SD Smith AR Goo L Broadly neutralizing activity of Zika virus-immune sera identifies a single viral serotype. Cell Rep (2016) 16:1485–91. 10.1016/j.celrep.2016.07.049 27481466 PMC5004740

[131] Kawiecki AB Christofferson RC. Zika virus-induced antibody response enhances dengue virus serotype 2 replication *in vitro*. J Infect Dis (2016) 214(9):1357–60. 10.1093/infdis/jiw377 27521359

[132] Mahalingam S Teixeira MM Halstead SB Zika enhancement: a reality check. Lancet Infect Dis (2017) 17(7):686–8. 10.1016/S1473-3099(17)30340-7 28653626

[133] Sasaki T Setthapramotebj C Kurosuaj T Nishimuraaj M Asaiaj A Omokokoaj MD Dengue virus neutralization and antibody-dependent enhancement activities of human monoclonal antibodies derived from dengue patients at acute phase of secondary infection. Antiviral Res (2013) 98(3):423–31. 10.1016/j.antiviral.2013.03.018 23545366

[134] Vaughn DW Green S Kalayanarooj S Innis BL Nimmannitya S Suntayakorn S Dengue viremia titer, antibody response pattern, and virus serotype correlate with disease severity. J Infect Dis (2000) 181(1):2–9. 10.1086/315215 10608744

[135] Halstead SB Observations related to pathogensis of dengue hemorrhagic fever. VI. Hypotheses and discussion. Yale J Biol Med (1970) 42(5):350–62. 5419208 PMC2591710

[136] Bardina SV Bunduc P Tripathi S Duehr J Frere JJ Brown JA Enhancement of Zika virus pathogenesis by preexisting antiflavivirus immunity. Science (2017) 356(6334):175–80. 10.1126/science.aal4365 28360135 PMC5714274

[137] Weiskopf D Sette A. T-cell immunity to infection with dengue virus in humans. Front Immunol (2014) 5:93. 10.3389/fimmu.2014.00093 24639680 PMC3945531

[138] Halstead SB Zompi S Protective immune responses to dengue virus infection and vaccines: perspectives from the field to the bench. Front Immunol (2015) 6:75 10.3389/fimmu.2015.00075 25741345 PMC4332367

[139] Wen J Ngono AE Regla-Nava JA Kim K Gorman MJ Diamond MS Dengue virus-reactive CD8+ T cells mediate cross-protection against subsequent Zika virus challenge. Nat Commun (2017) 8:1459. 10.1038/s41467-017-01669-z 29129917 PMC5682281

[140] Zellweger RM Eddy WE Tang WW Miller R Shresta S. CD8+ T cells prevent antigen-induced antibody-dependent enhancement of dengue disease in mice. J Immunol (2014) 193:4117–24. 10.4049/jimmunol.1401597 25217165 PMC4185219

[141] Zellweger RM Tang WW Eddy WE King K Sanchez MC Shresta S. CD8+ T cells can mediate short-term protection against heterotypic dengue virus reinfection in mice. J Virol (2015) 89:6494–505. 10.1128/JVI.00036-15 25855749 PMC4474296

[142] Halstead SB. Achieving safe, effective, and durable Zika virus vaccines: lessons from dengue. Lancet Infect Dis (2017) 17:e378–82. 10.1016/S1473-3099(17)30362-6 28711586

[143] Guzman MG Alvarez M Halstead SB. Secondary infection as a risk factor for dengue hemorrhagic fever/dengue shock syndrome: an historical perspective and role of antibody-dependent enhancement of infection. Arch Virol (2013) 158(7):1445–59. 10.1007/s00705-013-1645-3 23471635

[144] Guzmán MG Kourí G Valdés L Bravo J Vázquez S Halstead SB. Enhanced severity of secondary dengue-2 infections: death rates in 1981 and 1997 Cuban outbreaks. Rev Panam Salud Publica (2002) 11(4):223–7. 10.1590/S1020-49892002000400003 12049030

[145] Gibbons RV Kalanarooj S Jarman RG Nisalak A Vaughn DW Endy TP Analysis of repeat hospital admissions for dengue to estimate the frequency of third or fourth dengue infections resulting in admissions and dengue hemorrhagic fever, and serotype sequences. Am J Trop Med Hyg (2007) 77(5):910–3. 17984352

[146] Clark KB Onlamoon N Hsiao HM Perng GC Villinger F. Can non-human primates serve as models for investigating dengue disease pathogenesis? Front Microbiol (2013) 4:305. 10.3389/fmicb.2013.00305 24130557 PMC3795305

[147] Fibriansah G Tan JL Smith SA de Alwis R Ng TS Kostyuchenko VA A highly potent human antibody neutralizes dengue virus serotype 3 by binding across three surface proteins. Nat Commun (2015) 6:6341. 10.1038/ncomms7341 25698059 PMC4346626

[148] Munjal A Khandia R Dhama K Sachan S Karthik K Tiwari R Advances in developing therapies to combat Zika virus: current knowledge and future perspectives. Front Microbiol (2017) 8:Article 1469. 10.3389/fmicb.2017.01469 28824594 PMC5541032

[149] Munjal A Khandia R Tiwari R Chakraborty S Karthik K Dhama K Advances in designing and developing vaccines against Zika virus. Int J Pharmacol (2017) 7:667–76. 10.3923/ijp.2017

[150] Kam YW Lee CY Teo TH Howland SW Amrun SN Lum FM Cross-reactive dengue human monoclonal antibody prevents severe pathologies and death from Zika virus infections. JCI Insight (2017) 2(8):e92428. 10.1172/jci.insight.92428 28422757 PMC5396524

[151] Wang TT Sewatanon J Memoli MJ Wrammert J Bournazos S Bhaumik SK IgG antibodies to dengue enhanced for FcγRIIIA binding determine disease severity. Science (2017) 355(6323):395–8. 10.1126/science.aai8128 28126818 PMC5557095

[152] Woof JM Burton DR. Human antibody-Fc receptor interactions illuminated by crystal structures. Nat Rev Immunol (2004) 4(2):89–99. 10.1038/nri1266 15040582

[153] Larsson A Bålöw RM Lindahl TL Forsberg PO Chicken antibodies: taking advantage of evolution—a review. Poult Sci (1993) 72(10):1807–12. 10.3382/ps.0721807 8415358

[154] Fink AL Williams KL Harris E Alvine TD Henderson T Schiltz J Dengue virus specific IgY provides protection following lethal dengue virus challenge and is neutralizing in the absence of inducing antibody dependent enhancement. PLoS Negl Trop Dis (2017) 11(7):e0005721. 10.1371/journal.pntd.0005721 28686617 PMC5517069

[155] Jefferis R Lund J. Interaction sites on human IgG-Fc for FcgammaR: current models. Immunol Lett (2002) 82(1–2):57–65. 10.1016/S0165-2478(02)00019-6 12008035

[156] Schlothauer T Herter S Koller CF Grau-Richards S Steinhart V Spick C Novel human IgG1 and IgG4 Fc-engineered antibodies with completely abolished immune effector functions. Protein Eng Des Sel (2016) 29(10):457–66. 10.1093/protein/gzw040 27578889

[157] Armour KL Clark MR Hadley AG Williamson LM. Recombinant human IgG molecules lacking Fcgamma receptor I binding and monocyte triggering activities. Eur J Immunol (1999) 29(8):2613–24. 10.1002/(SICI)1521-4141(199908)29:08<2613::AID-IMMU2613>3.0.CO;2-J 10458776

[158] Shields RL Namenuk AK Hong K Meng YG Rae J Briggs J High resolution mapping of the binding site on human IgG1 for Fc gamma RI, Fc gamma RII, Fc gamma RIII, and FcRn and design of IgG1 variants with improved binding to the Fc gamma R. J Biol Chem (2001) 276(9):6591–604. 10.1074/jbc.M009483200 11096108

[159] Idusogie EE Presta LG Gazzano-Santoro H Totpal K Wong PY Ultsch M Mapping of the C1q binding site on rituxan, a chimeric antibody with a human IgG1 Fc. J Immunol (2000) 164(8):4178–84. 10.4049/jimmunol.164.8.4178 10754313

[160] Ramadhany R Hirai I Sasaki T Ono K Ramasoota P Ikuta K Antibody with an engineered Fc region as a therapeutic agent against dengue virus infection. Antiviral Res (2015) 124:61–8. 10.1016/j.antiviral.2015.10.012 26522769

[161] Shi X Deng Y Wang H Ji G Tan W Jiang T A bispecific antibody effectively neutralizes all four serotypes of dengue virus by simultaneous blocking virus attachment and fusion. MAbs (2016) 8(3):574–84. 10.1080/19420862.2016.1148850 26905804 PMC4966856

[162] Wang J Bardelli M Espinosa DA Pedotti M Ng TS Bianchi S A human bi-specific antibody against Zika virus with high therapeutic potential. Cell (2017) 171(1):229–41.e15. 10.1016/j.cell.2017.09.002 28938115 PMC5673489

[163] Hasan SS Miller A Sapparapu G Fernandez E Klose T Long F A human antibody against Zika virus crosslinks the E protein to prevent infection. Nat Commun (2017) 8:14722. 10.1038/ncomms14722 28300075 PMC5356071

[164] Lim SY Chan CE Lisowska MM Hanson BJ MacAry PA. The molecular engineering of an anti-idiotypic antibody for pharmacokinetic analysis of a fully human anti-infective. PLoS One (2015) 10(12):e0145381. 10.1371/journal.pone.0145381 26700297 PMC4689483

[165] Wong YH Goh BC Lim SY Teo EW Lim APC Dedon PC Structural mimicry of the dengue virus envelope glycoprotein revealed by the crystallographic study of an idiotype-anti-idiotype Fab complex. J Virol (2017). 10.1128/JVI.00406-17 28637753 PMC5553163

